# Evaluating the effect of Brainfood groups for people with mild cognitive impairment and mild dementia: preliminary mixed-methodology study

**DOI:** 10.1192/bjo.2018.29

**Published:** 2018-06-27

**Authors:** Saba Hassan, Elisa Aguirre, Anna Betz, Sarah Robertson, Deepak Sankhla, Claudia Cooper

**Affiliations:** UCL Department of Old Age Psychiatry, Division of Psychiatry, University College London, UK; Research and Development Department, North East London Foundation Trust (NELFT), Goodmayes Hospital, UK; Camden and Islington NHS Foundation Trust, Services for Ageing and Mental Health, Peckwater Centre, UK; UCL Department of Old Age Psychiatry, Division of Psychiatry, University College London, UK; North East London Foundation Trust, Redbridge Memory Service & Neuropsychological Pathway, Community Care Advice Centre, UK; UCL Department of Old Age Psychiatry, Division of Psychiatry, University College London and Camden and Islington NHS Foundation Trust, Services for Ageing and Mental Health, Peckwater Centre, UK

**Keywords:** Dementia, mild cognitive impairment (MCI), behaviour change

## Abstract

**Background:**

Brainfood is a 5-week group intervention for people with mild cognitive impairment and mild dementia, promoting cognitive health through a Mediterranean-style diet, exercise, mindfulness and health self-management.

**Aims:**

To evaluate Brainfood acceptability and the feasibility of conducting a randomised controlled trial; in a single group study in two National Health Service (NHS) memory services.

**Method:**

Participants self-completed quantitative and semi-structured questionnaires. Recruitment, attendance and outcome completion were the primary outcomes.

**Results:**

In total, 30 of 59 people invited to Brainfood attended; of the 26 (87%) who completed baseline measures: 25 (96%) completed post-intervention quantitative measures, 16 (67%) qualitative questions and 21 (81%) attended ≥3/5 sessions. Compared with baseline, participants reported significantly higher quality of life, Mediterranean diet adherence and exercising more, up to 2 months after the groups. Participants valued the groups and felt enabled to improve their well-being.

**Conclusions:**

Brainfood was acceptable and feasible to implement in an NHS setting.

**Declaration of interest:**

A.B. and C.C. developed Brainfood – they hold a creative commons license for the manual and make it available to use for free to all. The manual evolves iteratively, but the manual used in this research study is provided in an online supplement.

In total, 815 000 people live with dementia in the UK.^1^ The 2013 G8 Dementia summit identified preventative lifestyle changes as critical to the global dementia response: its prevalence would be halved if onset were delayed 5 years.^2^ The 2017 Lancet Commission on Dementia prevention, intervention and care encouraged researchers and policymakers to be ‘ambitious about prevention’; and suggested interventions enabling lifestyle change may have the potential to prevent or delay dementia. Memory services are potentially well placed to promote strategies to prevent dementia in people with mild cognitive impairment (MCI), and treat cognitive and behavioural symptoms in those already diagnosed. MCI is a heterogeneous state between normal ageing and early dementia. Its differentiation from dementia is primarily determined by the preservation of functional activities.^3^ It affects 19% of people aged 65 and over.^4^ Around 46% of people with MCI develop dementia within 3 years compared with 3% of the non-MCI population of the same age.^5^ More people are being diagnosed with MCI in Western countries as campaigns promote early presentation with memory problems to avoid crisis, but we know little about how to treat it. The National Institute for Health and Clinical Excellence recommends follow-up to ensure dementia is diagnosed and care planned at an early stage, but no specific treatments.^6^

## Lifestyle changes to prevent dementia

The USA National Institute of Health recommends intervention trials for dementia prevention encompassing multiple risk factors.^7^ In the Finnish Geriatric Intervention Study to Prevent Cognitive Impairment and Disability (FINGER) randomised controlled trial (RCT), an expert-led intervention that promoted healthy diet, increased physical activity, daily cognitive training, social engagement and monitoring and maintenance of metabolic and vascular factors, reduced cognitive impairment in people at increased dementia risk, with and without MCI.^7^^,^^8^ There is evidence that a high-fat diet leads to cognitive decline associated with decreased synaptic plasticity and decreased expression of cognitive-related proteins in hippocampi of rats;^9^ whereas in obese people with MCI, cognitive improvements are correlated with intentional weight loss.^10^ A meta-analysis has demonstrated improvements in cognition with aerobic exercise in healthy adults and people with MCI;^11^ and resistance training improved cognition in people with MCI in one RCT.^12^

Several mechanisms might explain the association between healthy lifestyle and better cognitive function. Diabetes and metabolic syndrome are associated with stroke and vascular problems. Impaired insulin receptor activation in Alzheimer's disease might explain the association between diabetes and Alzheimer's disease. Eating a Mediterranean-style diet (low in sugar and carbohydrates, meat and dairy; high in vegetables, fruit, fish, pulses and legumes) is associated with fewer vascular risk factors, and reduced plasma glucose and serum insulin levels, insulin resistance and markers of oxidative stress and inflammation.^13^ Exercise is neuroprotective; it promotes brain derived neurotrophic factor release, reducing cortisol and vascular risk.^14^

As the brain pathology that causes symptoms in dementia is inherently similar to that in MCI, people with mild dementia may also benefit from interventions promoting positive dietary and other lifestyle changes. Currently no evidence-based, lifestyle-focused interventions exist for people with dementia, nor are any such interventions available for people with MCI in a potentially cost-effective and scalable format. Addressing eating and drinking difficulties of people living with dementia was rated a research priority by people with dementia and their carers.^15^ People with dementia are more likely to be dehydrated and malnourished. The review, which included 43 controlled studies rated to be at high risk of bias, most situated in care homes, concluded that there is currently no good-quality evidence about how to improve intake. Social interventions (for example, promoting family-style mealtimes) were small but consistently indicated that such approaches improved autonomy, communication, mood, involvement and participation in meaningful activity in the context of food and drink.^16^ An intervention in people with dementia living in a Danish nursing home that involved oral nutritional supplements, oral care and group exercises improved energy intake and social and physical (although not cognitive) functioning relative to the control group.^17^

## Developing Brainfood intervention

We (A.B. and C.C.) developed Brainfood, a five-group session intervention promoting lifestyle change in 2013. We based the content on systematic review findings.^18^^,^^19^ After piloting the intervention within the memory service, we refined it using feedback from people with MCI and dementia and their family carers. The groups are offered to people with MCI and mild dementia attending several London National Health Service (NHS) memory services. Although the groups are based on best current evidence, there is no evidence regarding whether they successfully enable change in participants’ dietary and exercise behaviours or improve their well-being. We aimed to evaluate the acceptability of this low-intensity intervention and the feasibility of testing it in a pragmatic RCT. To address these aims, we set three primary objectives:

- to determine the proportion of people attending the groups completing baseline and follow-up measures and those attending at least three of five sessions;
- to measure change on our *a priori* primary outcome, EQ-5D quality of life score post-intervention; and
- to explore using qualitative methods whether and how attending Brainfood groups enabled positive lifestyle changes.

## Method

### Recruitment and procedures

Participants in this single group study were recruited from two memory services (inner and outer London). People diagnosed with MCI or dementia in the services were asked by clinicians if they would be interested in attending Brainfood groups. They were invited to do so alone, with a family carer or if more appropriate to their circumstances, the family carer attended on their behalf. Potential attendees were advised that the person who makes decisions about shopping and meal preparation for the person with cognitive impairment should attend. Taxis were provided at the outer London site for those who needed them, but this was not possible at the inner London site. Participants were given the dates of the groups in advance and a telephone reminder on the day of each group.

All questionnaires were administered by S.H.; S.H. was not involved in delivering the group sessions. People who joined the Brainfood groups at the first or second session were invited to take part in the research. Those declining participation in the research study were nonetheless invited to attend the groups. All research participants gave written, informed consent, then completed baseline measures immediately prior to the first session they attended. Participants were asked to complete outcome measures immediately after the final group session. S.H. visited participants at home, or the clinic if they preferred, to complete other post-intervention follow-up interviews, or completed the measures by phone where participants preferred this. Lack of capacity to take part in the study was an exclusion criterion, but no participants were excluded for this reason. Yorkshire and the Humber – Sheffield National Research Ethics Committee (16/YH/0456) approved the study.

### Quantitative measures

We recorded the proportion of people invited to Brainfood who attended and who took part in the study; whether participants attended alone or with a family member, or if the carer attended alone. At baseline, post-intervention and the 2-month follow-up the following measures were self-completed by participants, with family carer support in some cases, or by proxy if the carer attended alone.

- The EQ-5D: a reliable and valid visual analogue scale to measure health-related quality of life; lower scores indicate poorer quality of life.^20^
- The Geriatric Depression Scale: a 15-item, valid and reliable self-report assessment used to identify depression in older people.^21^
- A revised-short version of the Mediterranean diet score: a measure assessing the consumption of elements included in the Mediterranean diet, for example olive oil, wine, fruits, legumes and whole-grain intake. Low consumption of meat, coffee, commercial sweets and fizzy drinks is included and reverse scored. Higher scores indicate greater adherence to the diet. We used the short Mediterranean diet score, which has content, concurrent and predictive validity.^22^^,^^23^ In previous studies, it has been adapted for local diets: we added additional items on intake of commercial sweets, biscuits, cakes and pastries and sweetened and fizzy groups used previously.^24^ We evaluated content validity within the team and added two further items to ask participants whether they drank water with every meal and more than five cups of coffee daily.

We also asked participants to list the type, frequency and duration of exercise they undertook in the past week. We calculated the total number of hours of exercise. We asked about the number of alcohol units consumed in the preceding week. At follow-up we asked whether or not they had made any lifestyle changes because of Brainfood, with yes/no as possible responses.

We recorded, from clinical notes at baseline: diagnosis; whether antidementia medication was prescribed; whether or not they were attending cognitive stimulation therapy and their most recent score on Addenbrooke's Cognitive Examination III-Revised Version and the date of test; this assesses a range of cognitive domains and differentiates well between those with and without cognitive impairment.^25^

### Qualitative questionnaire

At follow-up the questionnaires included the open-ended questions: ‘What changes have you made because of Brainfood?’ and ‘Are there any other changes you have made?’

### Brainfood intervention

Brainfood comprises five group sessions held weekly or fortnightly at the memory services. Sessions are fully manualised and participants receive a manual to keep, and to record their personal goals. The groups were facilitated by: a social worker (A.B.) and a psychology graduate (S.R.) at memory service one; a health psychologist (E.A.) and clinical psychologist (D.S.) at memory service two. They covered: session one: eating regular meals, having a good breakfast, the importance of exercise; session two: good and bad carbohydrates and making social connections; session three: good and bad fats and looking after physical health; session four: Mediterranean diet and impact of alcohol; session five: putting it all together. All sessions involved tasting and sharing foods relevant to the sessions and included a brief mindfulness exercise that participants were encouraged to practice at home. Participants were guided to identify personal goals for behaviour change to try out between sessions; participants iteratively developed a personal action plan over the five sessions. The intervention manual is included as an online supplement (See Supplementary Document 1, available at https://doi.org/10.1192/bjo.2018.29).

### Analysis

We used SPSS version 24 to analyse quantitative data. We used descriptive statistics to summarise data. We reported the percentage of people invited to groups who attended, who took part in the study and completed outcomes; and the number of sessions attended. We assessed distribution of outcome variables and log-transformed data that was excessively skewed (skewness >1 or <−1). We used univariate paired *t*-tests to compare outcomes at baseline and follow-up; with last-observation-carried-forward to account for the one participant with missing follow-up data. Our sample size was based on the objective of providing estimates of intervention adherence and study recruitment for a future trial.

S.H., C.C. and S.R. reviewed participants’ responses to open-ended questions and undertook the qualitative analysis based on methodology described by Braun & Clarke.^26^ They identified themes independently that responded to the qualitative research question: what changes had the participants noted that they attributed to the group attendance? We used Pender's theory of health promotion as a framework for analyses, mapping identified themes to the components of this model: personal characteristics and experiences, perceived benefits and barriers to action, perceived self-efficacy and interpersonal and situation influences, and behavioural outcomes.^27^^,^^28^

## Results

### Study participation

A total of 59 potentially eligible people were invited to participate in Brainfood and expressed an initial interest during the study period. Thirty (51%) of these people attended the initial or second Brainfood session. All were screened as being potentially eligible and 26 (87%) agreed to take part in the study and completed baseline measures. The four attendees who did not take part arrived late and were either not invited to take part because S.H. had left the clinic or did not want to complete the questionnaires before the session because they would miss the group that was already underway. Of 26 participants at baseline 25 (96%) completed all post-intervention quantitative measures; one participant refused to complete either follow-up interview. Participation rates were broadly similar between memory service one and memory service two.

Of the participants, 17 (65%) attended sessions alone; 8 (31%) attended with a family member (7 with a spouse and one with an adult child), of whom 4 had a diagnosis of MCI and 4 had a diagnosis of dementia. For one participant, the family carer attended sessions alone, and proxy-completed the questionnaires on behalf of the person they were caring for who had dementia.

### Intervention participation

Groups took place between January and August 2017. In total, 21 of the 26 (81%) participants attended at least three out of the five sessions. Five people attended two sessions, four people attended three sessions, seven people attended four sessions and ten people attended five sessions. We recruited 14 (54%) of participants from memory service one (in which the participants took part in three cohorts each with five, three and six participants) and the remaining 12 (46%) from memory service two, who took part as one group.

### Participant characteristics

Thirteen (50%) of participants had a diagnosis of MCI; 12 (46%) had a diagnosis of dementia and one (4%) participant did not consent to us obtaining clinical information from medical records. For those for whom their Addenbrooke's Cognitive Examination III score was available in the last year before baseline, the mean score was 82.9 (s.d. = 10.2, *n* = 11) in participants with MCI and 79.7 (s.d. = 12.7, *n* = 10.2) in participants with dementia; the overall range of scores was 50–95. Thirteen (50%) participants were women. In total, 5 (19%) were attending Cognitive Stimulation Therapy at baseline; and 8 (31%) were taking antidementia medication at baseline.

### Quantitative outcomes

We log-transformed data on the amount of exercise taken and Geriatric Depression Scale scores, and resulting data was within our predetermined limits for conducting parametric analyses (skewness >−1 and <1) (Table 1).

**Table 1:** Scores for study outcomes

	Mean (s.d.)			Paired *t*-test (*P*)	
Outcome	Baseline(*n* = 26)	Post-intervention (*n* = 26)^a^	2-month post-intervention follow-up, (*n* = 26)^a^	Baseline/post-intervention comparison	Baseline/2-month post-intervention comparison
EQ-5D score	65.4 (17.5)	71.5 (13.9)	74.2 (12.9)	**2.7** (**0.013)**	**3.2** (**0.004)**
Mediterranean diet score	8.0 (2.7)	10.0 (2.1)	10.2 (2.1)	**3.4 (0.002)**	**3.5 (0.002)**
Geriatric Depression Scale score (log)	1.3 (0.7)	1.1 (0.8)	1.1 (0.7)	1.2 (0.23)	1.2 (0.23)
Exercise (hours per week) (log)	1.4 (0.7)	1.6 (0.9)	1.7 (0.8)	−1.6 (0.12)	**−2.7 (0.014)**

Log, log-transformed scores.

a. For one participant, we used last-intervention-carried-forward as they did not complete follow-up interviews.

Bold denotes significance *P* < 0.05.

### Well-being measures

EQ-5D scores increased between baseline and immediate post-intervention follow-up, and between baseline and follow-up 2 months post-intervention (Table 1). Geriatric Depression Scale scores did not change significantly over these time periods.

### Lifestyle measures

Post-intervention, 21 (84%) of participants with data reported that they had made changes because of Brainfood, and the same participants reported that they had sustained these changes 2 months later. Mediterranean diet scores increased significantly between baseline and post-intervention and 2-month follow-up, indicating greater adherence to Mediterranean diet over time (Table 1). In a *post hoc* analysis, greater mean change on the Mediterranean diet score was reported by people with dementia compared with those with MCI (3.7 *v.* 0.9) and by those attending with a carer (2.8 *v.* 1.5).

Walking, dancing and flexibility exercises were the main types of exercise reported. The amount of exercise reported increased from baseline to 2 months follow-up, but not between baseline and the immediate post-intervention. At baseline, 17 (65%) of participants drank no alcohol, 7 drank between one and ten units of alcohol a week, and two did not answer. None of the participants reported increased alcohol intake at subsequent post-intervention or 2-month follow-ups.

### Process evaluation and qualitative analysis

Of the 26 participants, 16 (62%) responded to the qualitative questions at the first follow-up and 12 of 21 (57%) responded to the qualitative questions at both follow-up points; with other respondents leaving the questions blank or writing ‘none’. Data saturation was attained with no new themes arising in the final three questionnaires analysed.

We identified nine subthemes, which we fitted within five themes informed by Pender's health promotion model: personal characteristics, perceived benefits to action, perceived self-efficacy, interpersonal and situation influences and behavioural outcomes. These are displayed in Table 2, with example quotes illustrating each theme, divided by diagnosis and discussed below.

**Table 2:** Summary of qualitative framework analysis results^a^ with example quotes

Model component^a^	Theme	People with dementia	People with mild cognitive impairment/diagnosis unknown
Personal experiences	Existing knowledge	‘It was good and however as mentioned before I had adopted this form of diet for many years‘(FU2)	‘I use 75% of the food recommended in the five sessions’ (FU1) ‘I've always eaten this way therefore not seeing many changes in myself however the groups were enjoyable and informative’ (FU2)
Perceived benefits of action	Awareness about healthy diet and impact on memory	‘Awareness of the interaction and impact of varied and healthy foods on memory and well-being’ (FU1)	‘Being educated about oils in particular and importance of variation in diet’ (FU1)
Perceived self-efficacy	Diet as an active choice	‘Understanding greatly increased of the choices to include in eating and the benefits they make… begin to try different pastas, breads, teas, oils, nuts, butters and increased awareness of various foods and their effects.’ (FU1)	‘More awareness of food intake’ (FU1) Using more oil, will work on using more grains, pulses, legumes and I will keep my 5 booklets and refer to them to enable me to correct my diet.’ (FU1)
	Increase in perceived control over health status		‘My attitude to what is still possible despite my medical condition’ (FU1)^b^ ‘My attitude has changed towards my health problems and I see how a healthier diet can help my sleep‘(FU2)^b^
Interpersonal and situation influences	Positive social effects of group	‘Thoroughly enjoyed meeting people who go through similar problems and learning about my problems in detail’ (FU2)	‘Nice meeting new people who undergo similar things some of us are still in contact’ (FU2)
Behavioural outcomes	Changing cooking/shopping habits	‘Enjoyed Mediterranean food throughout life but was nice to learn about how to incorporate it for home cooked meals.’(FU1) ‘Cooking a lot healthier now, enjoying trying out all the new herbs available from local market‘(FU2)	‘Familiarised myself with healthy food suppliers’ (FU1) ‘Have home cooked meals more often’ (FU2)
	Dietary changes	‘Cut down on sugar. Pumpkin seeds, nuts instead of sweets, cut down on sugar, much more lemon‘(FU1) ‘Using sweetener rather than sugar in tea, trying more teas’ (FU2) ‘Use more olive oil’ (FU2) ‘Using more oil, have been eating more grain based foods’	‘Increased oil intake slightly’ (FU1) ‘Stopping margarine. Starting olive oil, increasing intake of spinach, salad and nuts‘(FU1) ‘I cook with olive oil more, less sugar’ (FU2)
	Exercise	‘Joining the gym’ (FU1)	
	Mindfulness	‘Learning mindfulness. Helped with sleep practice.’ (FU1)

FU1, first follow-up; FU2, second follow-up.

a. Informed by Pender's health promotion model.

b. Same participant; other quotes within themes are from different participants.

#### Personal experiences

The only personal experience that was identified as influencing response to the groups was existing knowledge. Three participants (including people with MCI and dementia) indicated that the extent to which the sessions changed their behaviour was limited by the degree of accord between lifestyle changes suggested and their current practices.

#### Perceived benefits of action

Several of the participants reported that the groups increased their awareness of the perceived benefits of the targeted behaviour changes. This was most frequently discussed in relation to dietary change. One participant with dementia explained they were more aware of the ‘impact of varied and healthy foods on memory and well-being’ after attending the groups. A second participant with MCI reported increased awareness of the importance of ‘variation in diet’.

#### Perceived self-efficacy

Narratives of participants (at first (FU1) and second follow-up (FU2)) with MCI and dementia suggested that the groups had increased their understanding of diet as an active choice. One person with dementia spoke of how they understood more about the benefits of different food choices:

‘Understanding greatly increased the choices to include in eating and the benefits they make.’ (FU1)

A participant with MCI spoke of an intention to use the manuals as a resource when planning diet:

‘I will keep my 5 booklets and refer to them to enable me to correct my diet.’ (FU1)

One participant described (at both time points) how the groups had led to an understanding that despite having a cognitive disorder, they could still make changes that could improve their well-being:

‘My attitude to what is still possible despite my medical condition.’ (FU1)

‘My attitude has changed towards my health problems and I see how a healthier diet can help my sleep.’ (FU2)

#### Interpersonal and situation influences

Five participants, including people with dementia and MCI, reported enjoying or benefiting from the opportunity to meet together to share ideas, and the social aspect of the groups:

‘Wonderful workshop…thoroughly enjoyed meeting people who go through similar problems and learning about my problems in detail.’ (FU2)

This continued even after the 2-month follow-up, in which some participants decided to meet informally.

‘Nice meeting new people who undergo similar things, some of us are still in contact.’ (FU2)

#### Behavioural outcomes

Participants described making changes to how they ate, with participants with dementia and MCI making references to having more home-cooked meals and enjoying learning how to implement their understanding from the group in the meals they cooked:

‘Enjoyed Mediterranean food throughout life but was nice to learn about how to incorporate it for home cooked meals.’ (FU1)

One participant with MCI also described seeking out new places to buy food:

‘Familiarised myself with healthy food suppliers.’ (FU1)

Most (*n* = 15) participants described making and sustaining dietary changes recommended in the groups. For example, a participant with dementia described the following dietary changes:

‘Cut down on sugar. Pumpkin seeds, nuts instead of sweets, cut down on sugar, much more lemon.’ (FU1)

One participant mentioned ‘joining the gym’, and another described how mindfulness ‘helped with sleep’.

## Discussion

### Main findings

The Brainfood group intervention was accepted by participants, most of whom reported positive behaviour changes. Dietary change was most targeted in the groups, and most reported behaviour change was in this area. Facilitators of change appeared to be an increasing awareness of how the targeted lifestyle changes might improve their well-being, increased self-efficacy and awareness of diet being an active choice, which can influence well-being and positive social experiences in the group. In quantitative analyses, we found that reported Mediterranean-style dietary adherence, exercise and health-related quality of life increased compared with baseline up to 2 months follow-up post-intervention. Changes in depression scores over time were not significant but in the expected direction. Over half of the potential participants who expressed an interest in attending did so, and 80% of study participants attended three of five groups. As many faced barriers to attending including memory loss, physical illness and difficulties arranging transport (on one site), this is promising.

### Interpretation of our findings

Although our findings must be interpreted cautiously in this small, uncontrolled study, they suggest that the groups may have enabled meaningful lifestyle changes. This is the first study, to our knowledge, to evaluate an intervention to enable behaviour change in people with dementia and MCI, without a regular family carer acting as study partner. Even without a family carer to support implementation, people with mild dementia appeared able to use the sessions to make changes to lifestyle; our experiences suggested that the intervention manual was important in enabling this. We cannot determine the mechanisms, but previous studies report that Mediterranean diet^29^ and increased exercise^30^ are associated with increased quality of life, possibly mediated by improved physical, mental or social well-being or increased self-efficacy.^31^ The intervention involved supporting participants to make choices and personal plans for change. Perhaps this approach communicated that independence is possible despite memory loss. Participants valued the social experience. Where family carers attended, their experiences may have influenced the care recipients’ quality of life as family carer and care recipient quality of life are interlinked.^32^ We did not measure family carer quality of life and would do so in a future trial.

A systematic review of eight interventions to promote healthy diet in older people without cognitive impairment found that interventions involving more than one session and focusing on specific changes, for example increasing fruit and vegetable intake were more effective. Only three of the interventions reviewed were explicitly based on a health promotion theory; these included Pender's health promotion model.^27^ In our framework analysis based on Pender's model, we identified that perceived benefits, self-efficacy and interpersonal relationships were likely drivers of the behavioural outcomes we recorded. Pender's model also includes barriers to behavioural change, but we did not identify description of barriers to action in the participants’ responses. Perhaps people with dementia or MCI attending groups and responding to the open-ended questions experienced fewer barriers to implementing recommended changes than those who did not attend or respond, or perhaps our open-ended questions did not adequately prompt participants to report them.

### Strengths and limitations

This was an exploratory, pilot study designed to work within current NHS service provision, with broad inclusion criteria and brief outcome interviews. There was no control group. Outcome measures were self-completed, but may have been influenced by social desirability bias. Some participants attended with family members who were usually present when they completed measures and may have influenced ratings or assisted recall. In one case, a family carer attended alone and proxy completed the measures. There are systematic differences in how family carers and people living with dementia rate quality of life,^33^ so this is a limitation. We think that opening this trial to people with and without a family carer was also a strength, as people with dementia without a family carer are rarely included in research developing interventions designed for them to use.^34^ Although the follow-up period was too brief to evaluate changes in cognitive function, this would be an important outcome in a future trial, as higher Mediterranean dietary adherence is associated with a reduced dementia risk.^35^

### Implications

Our intervention was conceived because people with mild dementia and MCI attending our memory services wanted more information about how lifestyle changes might improve their symptoms, so we included both these diagnostic groups in the study. The Mediterranean diet score has been used in populations with cognitive impairment,^23^ but not in people with mild dementia. Participants with mild dementia had similar cognitive function to those with MCI, but a more homogenous population would arguably be preferable in a future pragmatic study as mechanisms for enabling behavioural change may differ in people with dementia who were by definition experiencing functional impairment. In our *post hoc* analysis, Mediterranean diet adherence increased more in people with mild dementia compared with those with MCI. We focused the intervention on how to help cognitive symptoms rather than on diagnosis. Perhaps participants with mild dementia particularly benefited from this approach because promoting self-efficacy and independence challenges the commonly held belief that as cognitive decline in dementia is inevitable, it is too late to make positive life changes. Perhaps people with dementia were more highly motivated to change, or their carers were more motivated to support them to implement changes.

Brainfood groups were offered in additional to all available usual care. There are no other groups routinely available in either trust that specifically promote Mediterranean dietary adherence or exercise, which were our main outcomes of behavioural change, so we do not think that attendance at other groups could have accounted for the changes we saw on these measures. We recorded and report attendance at cognitive stimulation groups at baseline, as these were the only groups offered (only to people with dementia) within the clinical services involved in the study during the study period.

In conclusion, this pilot study demonstrated that Brainfood, a psychosocial intervention to promote healthy diet, exercise and mindfulness in people with MCI and mild dementia was feasible to implement in a real-world NHS setting. Dietary change was most targeted in the groups, and most reported behaviour change was in this area. Facilitators of change appeared to be an increasing awareness of how the targeted lifestyle changes might improve their well-being, increased self-efficacy and awareness of diet being an active choice, which can influence well-being and positive social experiences in the group. Evaluation of the intervention in a randomised controlled trial is now planned.

## Supplementary material

For supplementary material accompanying this paper visit http://dx.doi.org/10.1192/bjo.2018.29. click here to view supplementary material
