# Supplementary material for: Evaluating the effect of Brainfood groups for people with mild cognitive impairment and mild dementia: preliminary mixed-methodology study
Source: BJPsych Open. 2018 Jun 27;4(4):208–14. doi: 10.1192/bjo.2018.29 (PMC6034463; doi:10.1192/bjo.2018.29)
Supplement: Supplementary file 1 [file S2056472418000297sup001.pdf]

Brain food

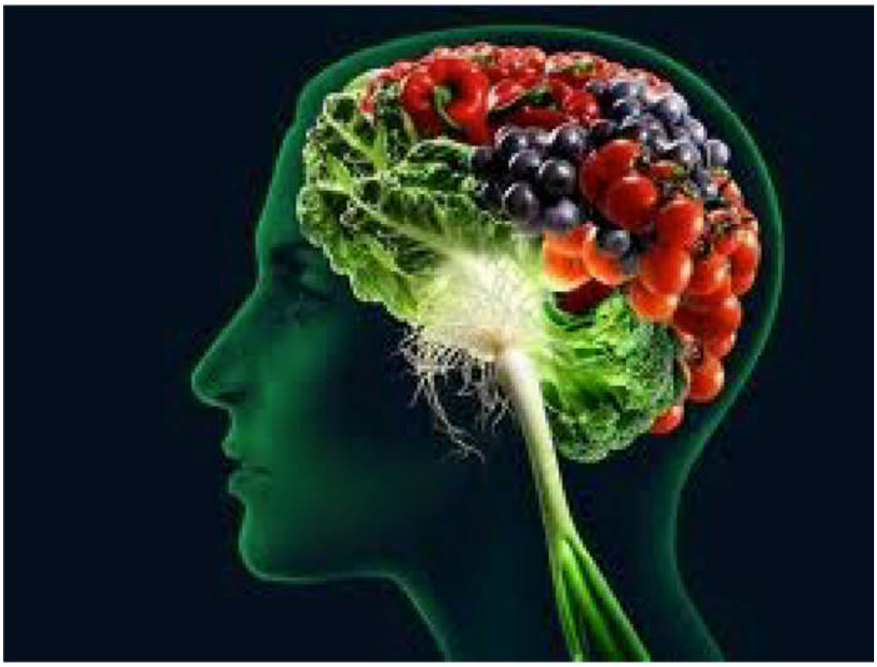

by Dr.Claudia Cooper & Anna Betz
Camden Memory Service
anna.betz@candi.nhs.uk

Session One

Attribution-NonCommercial-ShareAlike CC BY-NC-SA

Contents

Session 1: Starting well

Session 2: Good and bad carbohydrates

Session 3: Good and bad fats

Session 4: The Mediterranean diet

Session 5: Vitamins, minerals and putting it all together

Reunion session: a month after the course ends

Attribution-NonCommercial-ShareAlike CC BY-NC-SA

Introduction

What we eat and drink affects our physical and mental health.

The food that we eat provides fuel for our brain. Low quality fuel can make memory worse and high quality fuel can make it better. It's never too late to provide high quality fuel for our brains.

You are here because you or someone you help with food and cooking has memory problems. There is no cure or magic bullet for memory problems, but we can help. Things that can help memory include:

- Medication for some types of dementia
- Cognitive stimulation therapy
- Staying active
- Eating a healthy diet
- Drinking plenty of fluids
- Enjoying social activities
- Looking after physical health
- Having regular meals

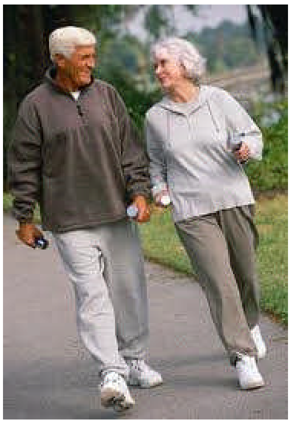

We can't promise that things will get better, but hope to give you some ideas about how changing what you eat, drink and do might help.

We will cover some strategies which help many people. You are probably doing some already.

Attribution-NonCommercial-ShareAlike CC BY-NC-SA

Beginning

Now let's introduce ourselves.

We will be talking mainly about food and activities but you or others might want to share personal stories with the group. Please respect confidentiality of the group. What is said in the group stays in the group.

Attribution-NonCommercial-ShareAlike CC BY-NC-SA

Here is an outline of what the programme covers:

Today is an introduction, in which we will discuss breakfast, timings of meals and exercise as possible ways to help memory.

Here is what we will cover in the other sessions:

Session 2: Good and bad carbohydrates
Session 3: Good and bad fats
Session 4: Mediterranean diet
Session 5: Putting it all together

This booklet is for you to keep and at each session we will give you more information covering what we talk about.

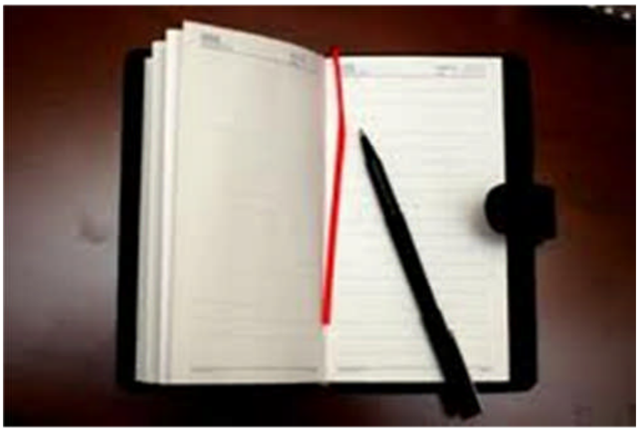

Attribution-NonCommercial-ShareAlike CC BY-NC-SA

Session 1: Starting well

Session Plan

1. Introducing the Course
2. Making meals interesting
3. What to eat for breakfast
4. When to eat
5. Eating together
6. Summary and discussion

Attribution-NonCommercial-ShareAlike CC BY-NC-SA

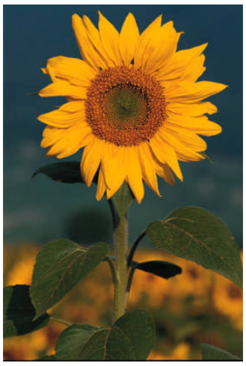

Introduction

- Everyone needs the right nutrients to live well.
- Diet can affect energy levels and ability to think clearly. A healthy diet also improves physical health, which is linked to memory.
- It is important that food is enjoyable. There are lots of different ways to eat healthily and we want to find ways that interest you.

Tell us what foods you and the person you care for enjoy? What makes eating enjoyable?

Attribution-NonCommercial-ShareAlike CC BY-NC-SA

Breakfast

- What do you/ the person you care for usually have for breakfast?

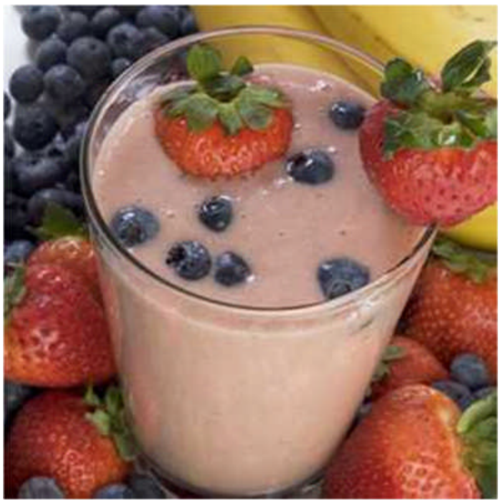

Attribution-NonCommercial-ShareAlike CC BY-NC-SA

- The first meal of the day is important for a good start.
- Oats or oatmeal make a good breakfast because energy is released slowly.
- It’s good to have some protein to keep your energy levels up, for example
  - Pumpkin seeds, walnuts, pecan or other nuts on cereal.

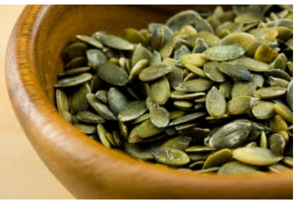

  - Salmon with tomatoes

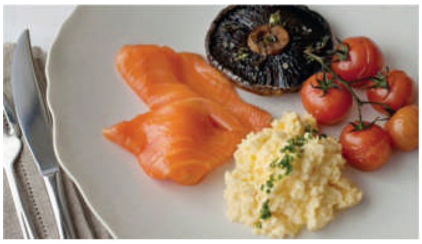

  - Humours

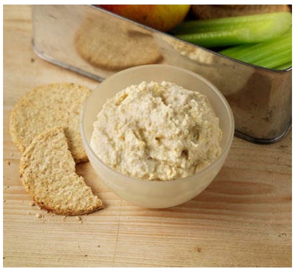

  - Beans, lentils

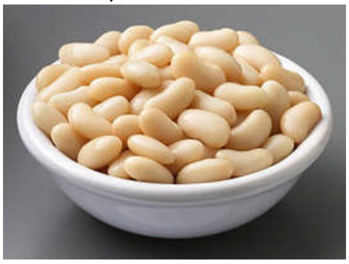

  - Eggs

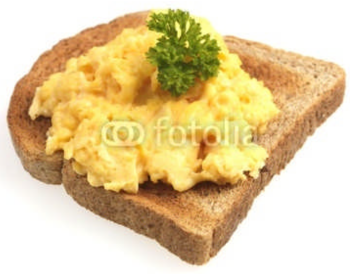

  - Avocado

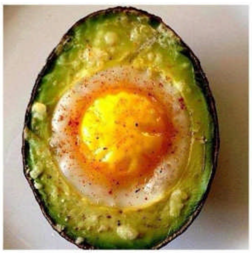

Attribution-NonCommercial-ShareAlike CC BY-NC-SA

Food to share

Every week we will bring some food for you to try. This week we have some suggestions for a healthy breakfast and healthy snacks.

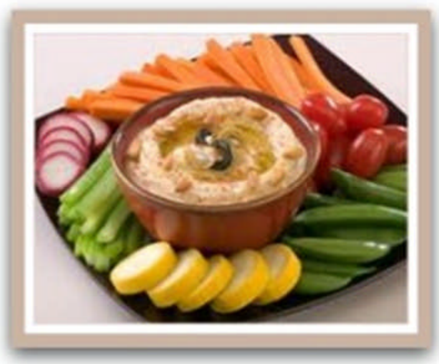

While we try these we can discuss what changes you might want to try to your usual breakfast.

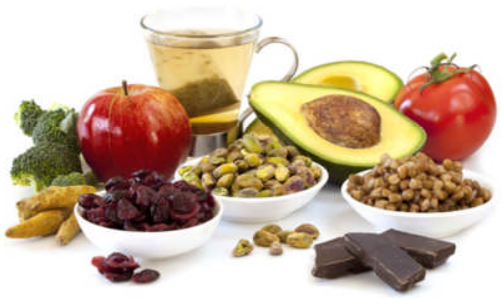

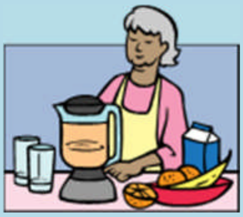

Breakfast smoothie recipe

Oatmeal

Almond, coconut, hemp seed or oat milk

Nuts and seeds

Some red fruit: strawberries, blueberries, raspberries You could add small amounts of other fruit: kiwis, apple, half a banana

Attribution-NonCommercial-ShareAlike CC BY-NC-SA

When to eat

When do you usually eat your meals?<br><br><br><br>What do you eat for snacks between meals?<br><br><br><br>What works well for you that you could share with others?

Attribution-NonCommercial-ShareAlike CC BY-NC-SA

Meal timings

Like the well known saying: Breakfast like a king, lunch like a prince, dinner like a pauper!

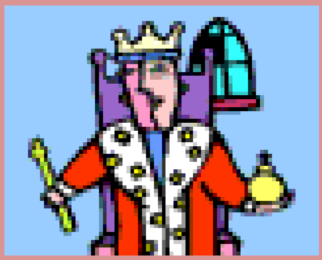

- It is important to start with a good breakfast
- Eat at regular times
- Try to avoid eating too late.

Would you like to make changes to your meal times?

Is there anything that would help you make changes?

Attribution-NonCommercial-ShareAlike CC BY-NC-SA

Eating together

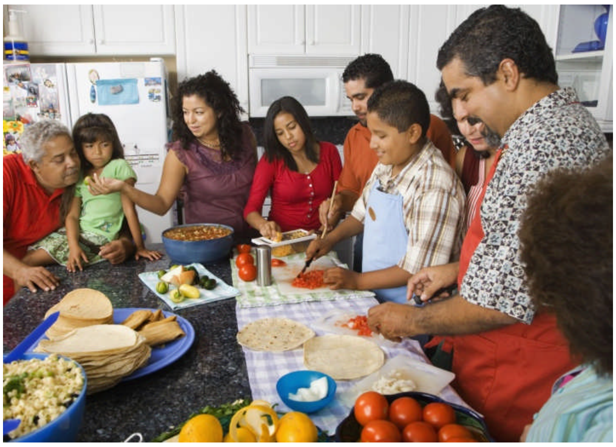

Where and with whom do you usually eat your meals?

In what circumstances do you most enjoy eating?

What changes might you want to make to this?

Attribution-NonCommercial-ShareAlike CC BY-NC-SA

Exercise

People who exercise more have better memories.

- Exercise improves cardiovascular health, which is strongly linked to memory problems.
- It probably also reduces inflammation in the brain and buildup of a protein called amyloid. Inflammation and amyloid both happen in Alzheimer’s disease.
- Exercise often includes social contact too, which is good for the brain.

Exercise can be difficult for people with physical health problems, but there is always some exercise that is possible.

What exercise do you do regularly? Do you enjoy it?

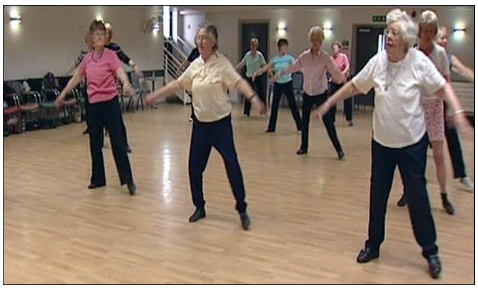

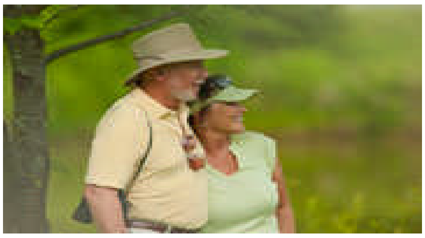

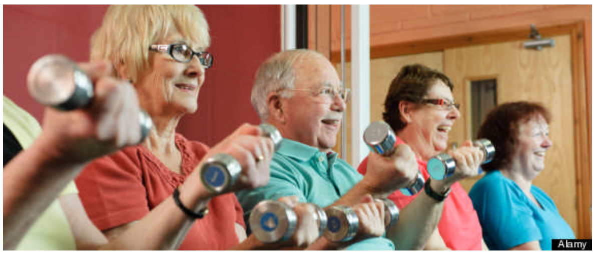

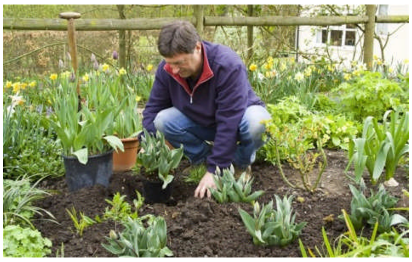

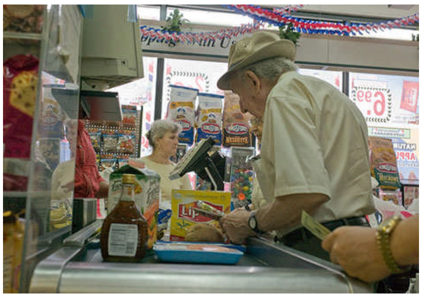

Attribution-NonCommercial-ShareAlike CC BY-NC-SA

Sleep & Relaxation

Sleep plays an important role in memory, both before and after learning a new task. Lack of adequate sleep affects mood, motivation, judgment, and our perception of events.

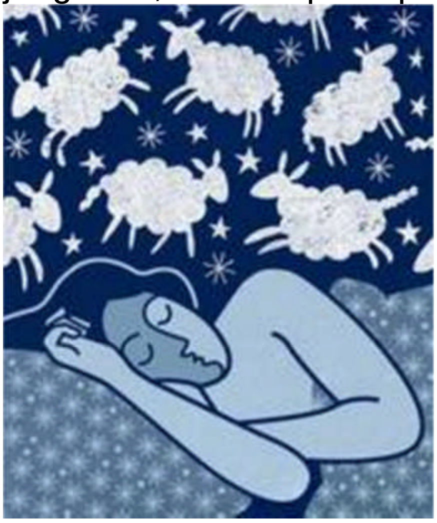

Here are some tips for a good night's sleep:

- Set a specific time for getting up each day.
- Don't take a nap during the day.
- Take daily exercise and spend time outside– daylight helps.
- Stop drinking tea and coffee for a few hours before bedtime.
- Avoid drinking alcohol and smoking.
- Don't eat a big meal just before bedtime.
- Create a relaxing bedtime routine, such as taking a bath, listening to soft music, and drinking a warm, milky drink.
- Talk to your doctor if you are worried about your sleep. Some people have also been helped by complementary medicines such as **Valerian, Passion flower, Hops, Oats, Borage, Leon Balm, Lavender and Roseroot. Like medicines, all have some possible side effects and interact with some medicines – here is a helpful website:**

http://www.rcpsych.ac.uk/healthadvice/treatmentswellbeing/complementarytherapy.aspx

Attribution-NonCommercial-ShareAlike CC BY-NC-SA

Making changes

Write down what changes you will try in the next couple of weeks.

Changes to breakfast<br><br><br><br>Changes to meal times<br><br><br><br>Exercise and any other changes

Attribution-NonCommercial-ShareAlike CC BY-NC-SA

Mindfulness Practice: 3 mins coping with difficulty space

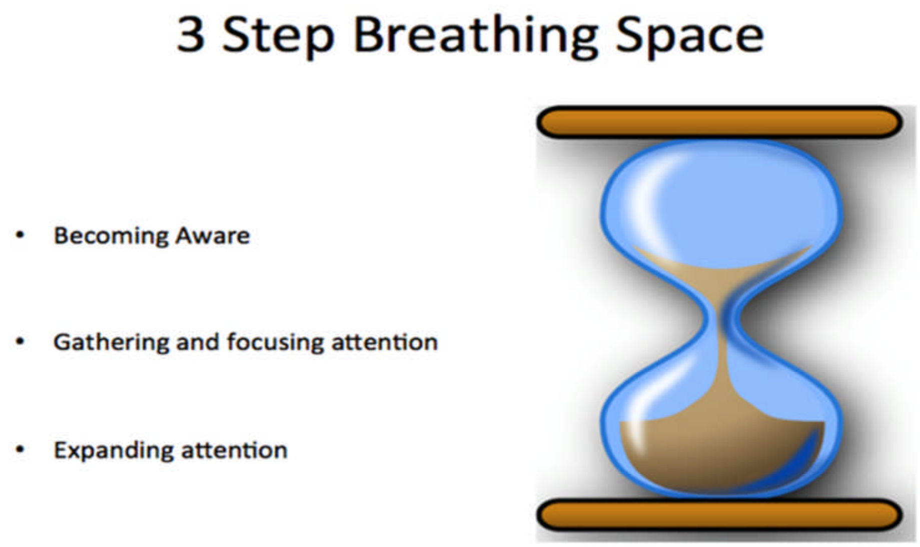

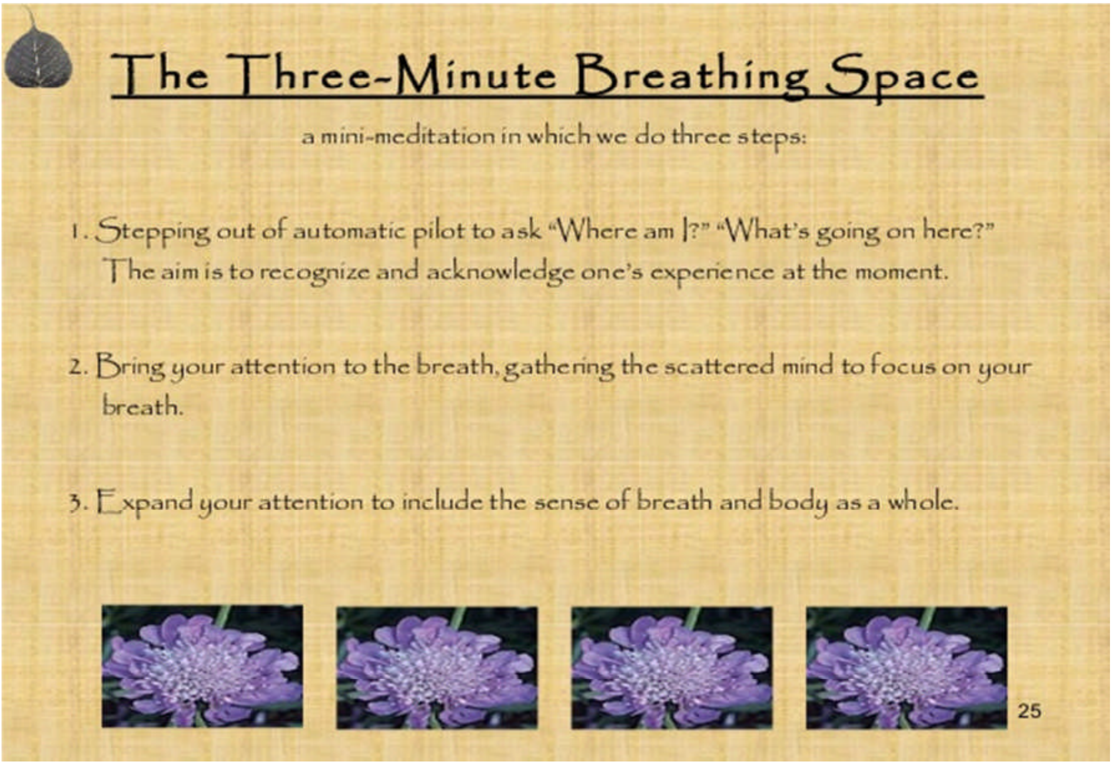

Attribution-NonCommercial-ShareAlike CC BY-NC-SA

Summary

Today, we have talked about:

- Introduction to the Course
- Making meals interesting
- What to eat for breakfast
- When to eat
- Eating together
- Taking exercise

FOR NEXT SESSION

Complete the food diary about what changes you have made and if this has made a difference to you

When do you think you might have the opportunity to make these changes?

What might get in the way?

Attribution-NonCommercial-ShareAlike CC BY-NC-SA

Brain Food

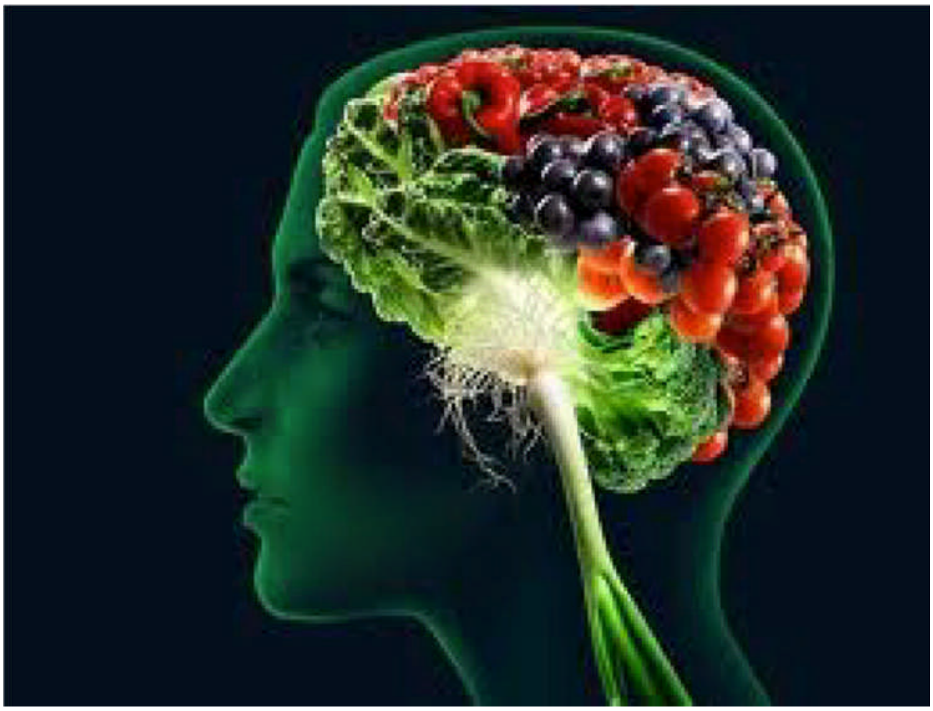

by Dr. Claudia Cooper & Anna Betz
Camden Memory Service
anna.betz@candi.nhs.uk

Session two

Attribution-NonCommercial-ShareAlike CC BY-NC-SA

Session 2: Good and bad carbohydrates

Session Plan

- Review of last week
- Why complex carbohydrates are good for your brain
- What are complex carbohydrates?
- Social connections
- Planning changes

Attribution-NonCommercial-ShareAlike CC BY-NC-SA

What changes did you try last week?

Last week we talked about:

Breakfast

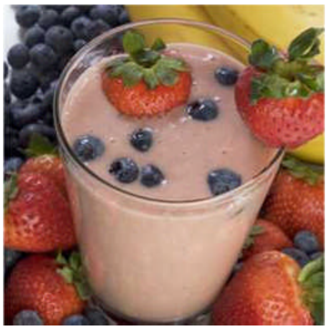

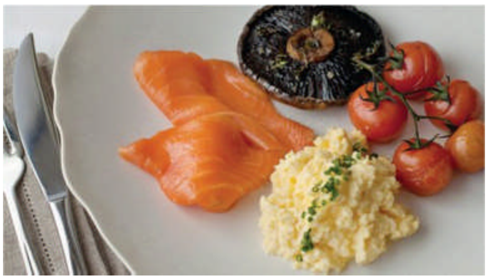

Mealtimes and snacks

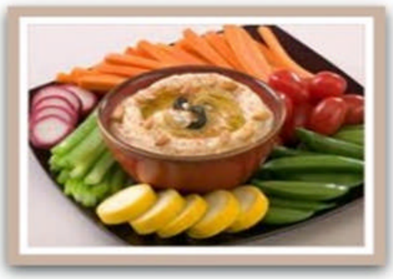

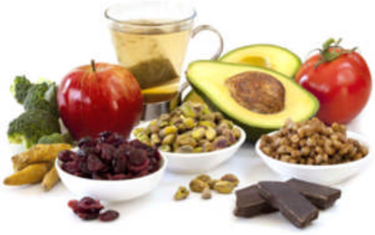

Exercise

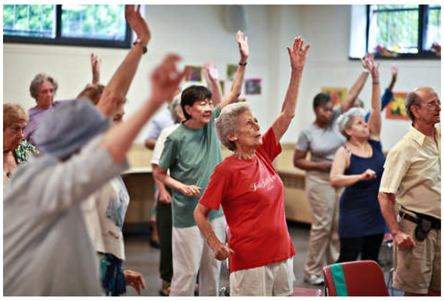

Discussion

What went well?
Were there problems?
What changes will you be continuing?

Attribution-NonCommercial-ShareAlike CC BY-NC-SA

Carbohydrates

- The pictures below are all of carbohydrates.
- We'll start by thinking about refined/simple and unrefined/complex carbohydrates.

**Refined/simple carbohydrates**	**Unrefined/complex carbohydrates**
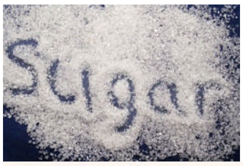 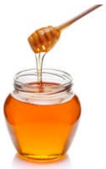 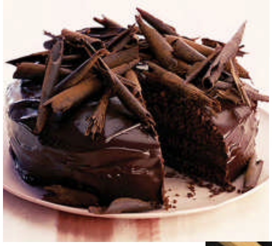 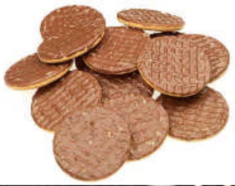 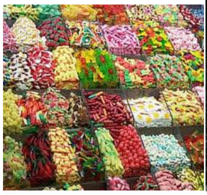 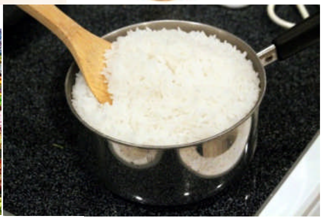 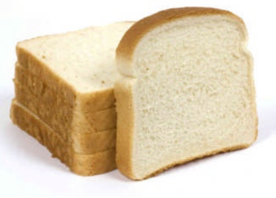 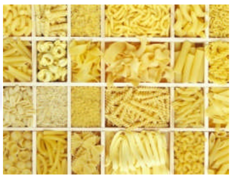	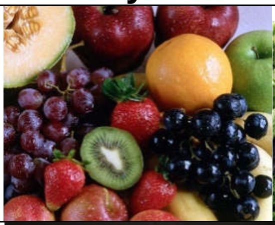 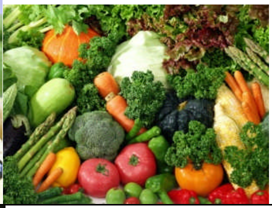<br>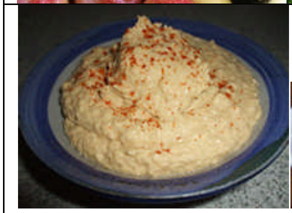 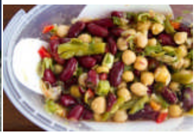<br>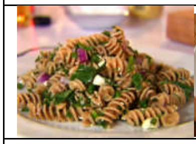 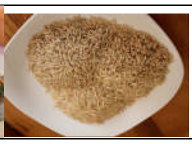<br>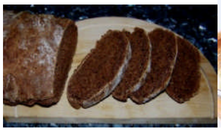 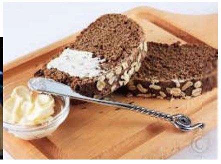<br>Wholemeal, pumpernickel, rye or spelt bread

Why does it make a difference?

Attribution-NonCommercial-ShareAlike CC BY-NC-SA

Some types of carbohydrates are better for the brain than others.

We digest carbohydrates to make glucose, which is our fuel.
Our bodies need insulin to use this fuel.

Eating carbohydrates with a high glycaemic load causes spikes in blood glucose followed by spikes in insulin.

Eating low glycaemic load carbohydrates causes a more gradual rise in glucose and less sudden release of insulin into the blood stream. This is better for the brain.

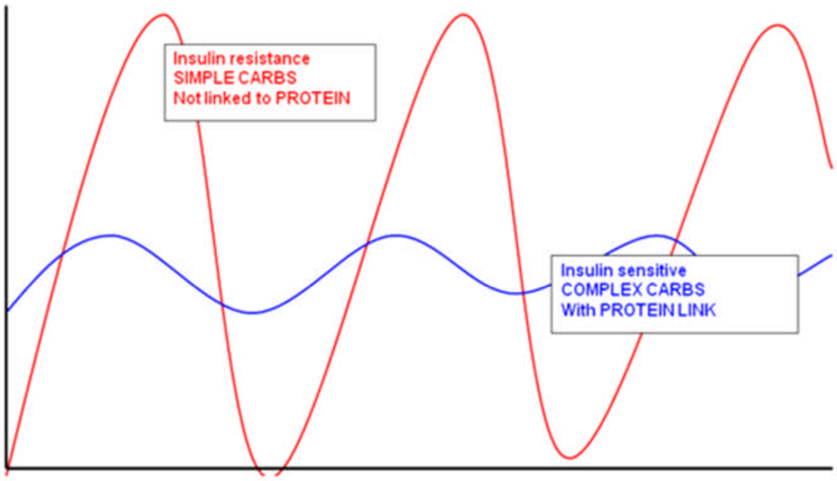

The brain uses more fuel than any other part of the body, and works better with a steady fuel supply. Many people have found that avoiding glucose and insulin spikes is good for the brain. You can do this by

- **choosing carbohydrates with a low glycaemic load**
- **Combining carbohydrates with some protein**
- **eating less carbohydrates overall**

Vegetables and fruits

Attribution-NonCommercial-ShareAlike CC BY-NC-SA

A brain-healthy diet means plenty of fruits and vegetables. They are packed with vitamins and minerals, and a good source of fibre especially when eaten raw.

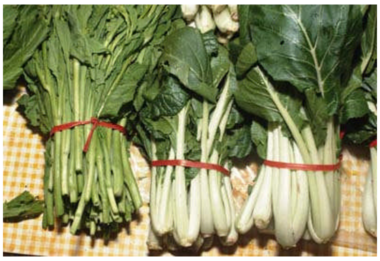

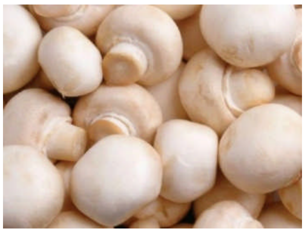

Green leafy vegetables are particularly good for memory as they are rich in folate, and contain antioxidants, which naturally reduce inflammation. Inflammation occurs in the brain in Alzheimer's disease.

Smoothies are an excellent way to consume plenty of raw vegetables. Celery makes a nutrient rich ingredient in a green smoothie. It mixes well with spinach and fresh lemon and helps to reduce inflammation.

Green smoothie

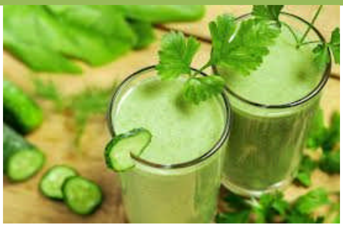

- 1 cup of raw spinach
- 1 cup of chopped raw celery
- ½ apple with skin
- ½ avocado
- Fresh juice of 1 lemon
- Small amount of fresh ginger
- Additional options: fresh parsley, coriander, cucumber, red pepper, kiwi ….
- Cover well with water and blend

Attribution-NonCommercial-ShareAlike CC BY-NC-SA

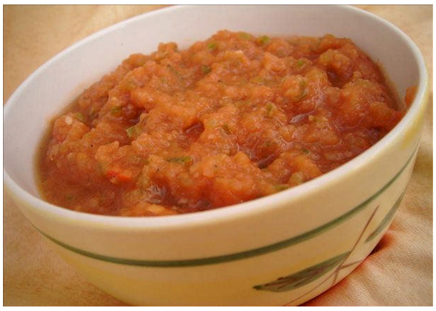

This is a tomato sauce, traditionally eaten in Spain. Tomatoes, onions or leeks, garlic, green peppers and olive oil are sautéed in a frying pan. It can be eaten with brown rice or eggs, or used as an ingredient in other dishes.

One chopped onion or leek
Several chopped tomatoes or half a tin of plum tomatoes
A chopped green pepper
2 cloves garlic, minced

Fry these ingredients in olive oil. Add 1/4 teaspoon ground coriander, oregano and fresh pepper to taste.

Guacamole

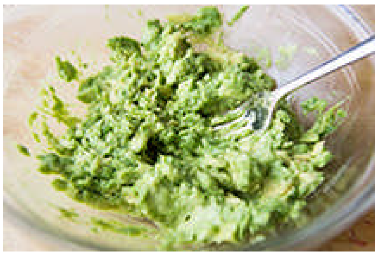

Guacamole is a dip made from avocados. All you really need for guacamole is ripe avocados and salt. After that, a little lime or lemon juice. Add chopped cilantro, chives, onion, and tomato, if you want.

Ingredients

- 2 ripe avocados
- 1/2 teaspoon salt
- 1 Tbsp of fresh lime juice or lemon juice
- 2 Tbsp of minced red onion or thinly sliced green onion
- 2 tablespoons cilantro (leaves and tender stems), finely chopped
- A dash of black pepper
- 1/2 ripe tomato, seeds and pulp removed, chopped
- Garnish with red radishes or jicama.

Attribution-NonCommercial-ShareAlike CC BY-NC-SA

Hummus

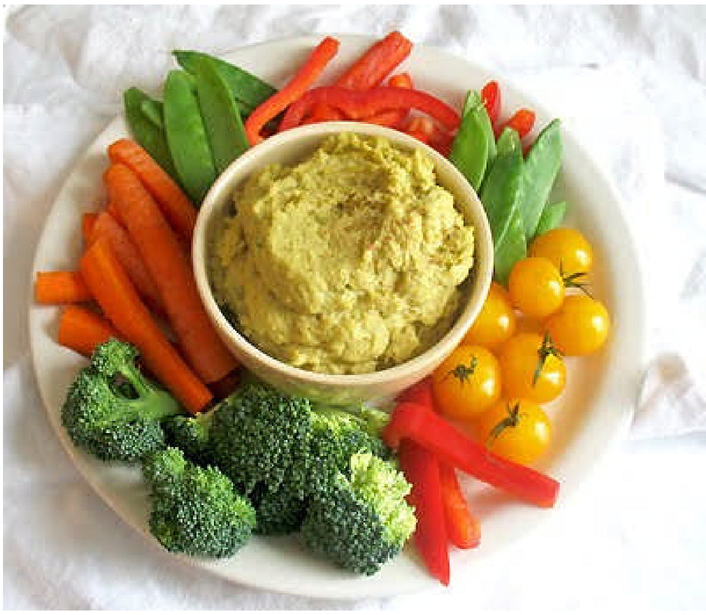

Hummus is great with nearly any raw chopped vegetable, as a spread or as a side dish with burgers, sausages or fish. Serve topped with a drizzle of extra virgin olive oil or chopped parsley. You can add chopped olives or sun-dried tomatoes.

Basic Hummus Recipe

- **Two tins of cooked chickpeas**
- **3 garlic cloves, peeled**
- **100ml tahini (also called sesame seed paste)**
- **50-100ml cup fresh squeezed lemon juice**
- **100ml Extra Virgin Olive**
- **2 tablespoons of Flax seed oil**
- **100ml water, or more as needed**
- **salt to taste**

In a food processor, chop the garlic cloves. Add tahini, lemon juice and water, process until smooth.

Add drained chickpeas and cayenne to the mixture. Process until well blended while adding additional water, as needed. Add salt to taste. Once blended, process another minute or so to add air to the hummus.

Attribution-NonCommercial-ShareAlike CC BY-NC-SA

Satisfying low carbohydrate meals

Here are some ideas for meals that are low in carbohydrates. What low carbohydrate meals do you enjoy?

- Salad with grilled chicken or fish

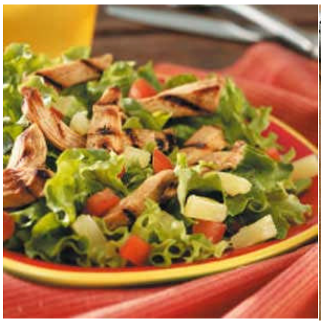
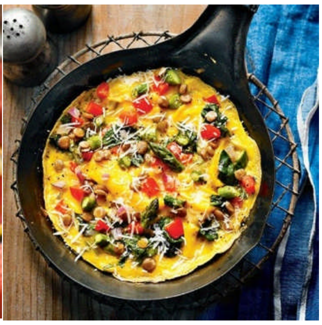

- Vegetable stir-fry with small amounts of seafood or lean meat
- Adding chopped, dark green, leafy vegetables or leftover vegetables to an omelette
- Scrambled eggs with mushroom and spinach can make a healthy breakfast or small lunch.
- Steam or sautée vegetables, with a little seasoning or olive oil and fresh lemon, or a dip like made with aubergine or avocado
- Roast or grill your veggies with salt, pepper, and a little olive oil. E.g. peppers, aubergine, courgettes
- Putting vegetables in soups including miso soup with some fresh ginger helps digestion and stimulates appetite.

Attribution-NonCommercial-ShareAlike CC BY-NC-SA

Fruits

The healthiest fruits are those low in sugar. These are:

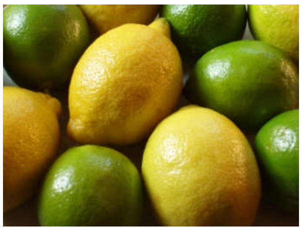
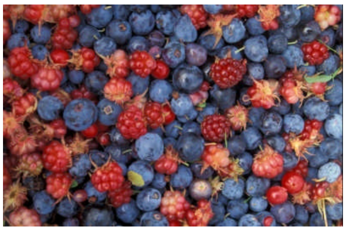
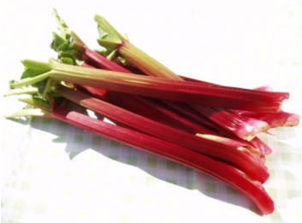

- Lemon or Lime
- Rhubarb
- Raspberries
- Blackberries
- Cranberries

**Low to medium sugar fruits (good in moderation)**	**Fairly high in sugar**	**Very high in sugar**
• Strawberries<br>• Blueberries<br>• Papaya<br>• Watermelon<br>• Peaches<br>• Nectarines<br>• Cantaloupes<br>• Apples<br>• Apricots (fresh)<br>• Grapefruit<br>• Cherries (sour)<br>• Guavas	• Plums<br>• Kiwifruit<br>• Pears<br>• Pineapple	• Oranges<br>• Tangerines<br>• Grapes<br>• Pomegranates<br>• Mangos<br>• Figs<br>• Bananas<br>• Dried Fruit, such as dates, raisins, dried apricots, and prunes

What fruits do you eat most frequently? Are there changes you might make to this?

Attribution-NonCommercial-ShareAlike CC BY-NC-SA

Healthier carbohydrates

Which of these do you have now?
Which others might you try?

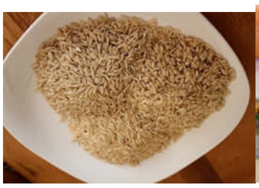
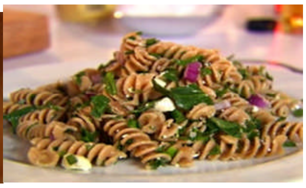
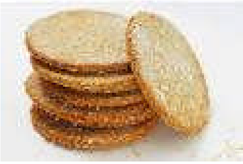
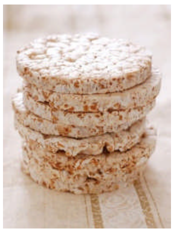

Wholemeal rice **Wholemeal pasta** **Oatcakes** **rice cakes**

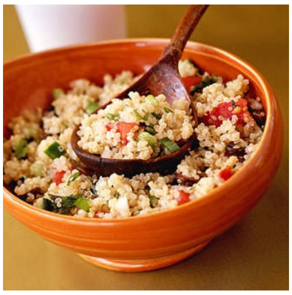

Quinoa: tasty and a good source of protein, iron, and fibre; rinse it then cook for about 20 minutes

Mix with steamed or raw vegetables and add a little salad dressing to taste. Eat hot or cold.

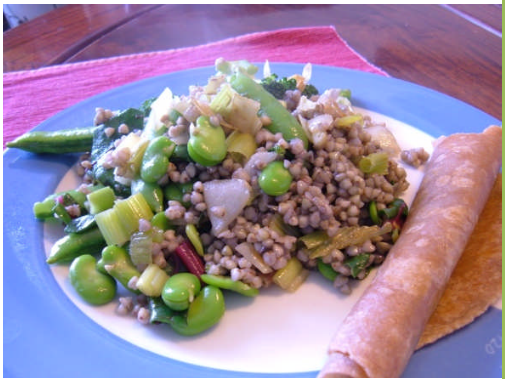

Buckwheat: A Good Source Of fibre. Wheat-free!

Boil three cups of water. Add some salt. Add two cups of buckwheat and stir. As soon as the grain expands and soaks up the water at the top, cover with the lid and leave it on the lowest setting possible for 20-30 minutes or until all the water is absorbed.

You can add fried onions, mushrooms, vegetables, beans and cold pressed vegetable oils.

Attribution-NonCommercial-ShareAlike CC BY-NC-SA

Legumes

These include beans, peas, lentils and peanuts. They are:

- Excellent source of protein.
- Good source fiber.
- Low in fat.
- A good source of folic acid, phosphorus, potassium, iron, zinc, calcium, and selenium.
- Rich in B vitamins and antioxidants.
- Low Glycemic Index

You can add them to soups, casseroles and meat sauces to add extra texture and flavour.
Which of these do you eat already and which might you try? Do you have recipes/ideas to share?

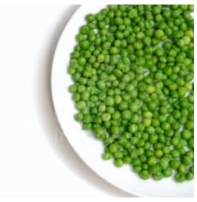
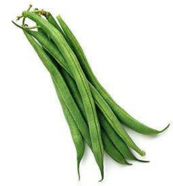
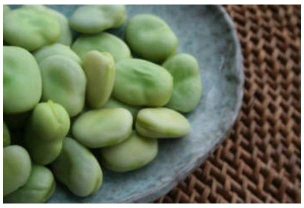

Garden peas **runner beans** **broad beans**

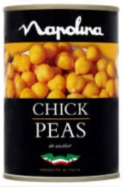

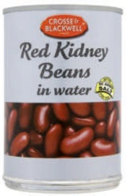

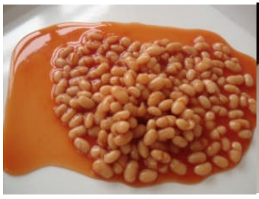
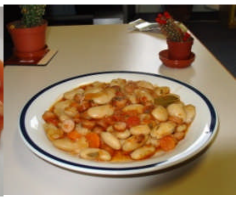
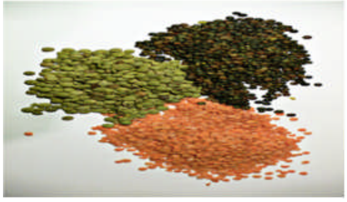

Chick peas, kidney beans, baked beans (without sugar)**, butter beans, broad beans, lentils.**

Attribution-NonCommercial-ShareAlike CC BY-NC-SA

How much?

Every day we recommend:

2+ vegetable servings. Raw vegetables are particularly good for you.

3+ fruit portions (including natural fruit juices)

Every week:

3+ servings of legumes a week

2+ servings of Sofrito (sauce made with tomato and onion, leek or garlic and simmered with olive oil)

But try to avoid:

- Sugary drinks
- Commercial bakery goods, sweets, and pastries
- Spread fats
- Red and processed meats (have <1 serving/day)

Attribution-NonCommercial-ShareAlike CC BY-NC-SA

Making connections

People with memory problems who are socially active tend to have better memories for longer than those who have memory problems but don't have regular social activities. This might be because:

- They are less likely to become depressed or anxious, or recover more quickly if they do
- Social contact is stimulating and enjoyable

There are lots of social groups doing varied activities in Camden. We have brought some information to show you about current groups.

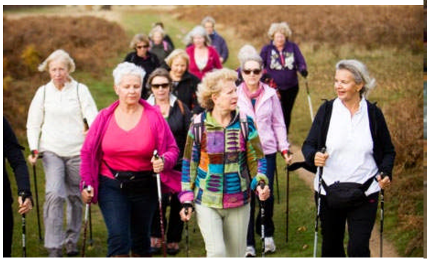

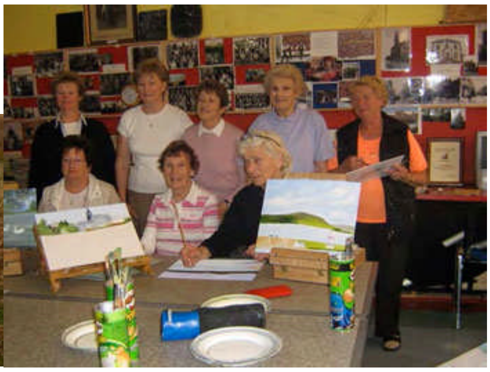

What groups do you attend now?
Which other groups might you be interested in going to?

Attribution-NonCommercial-ShareAlike CC BY-NC-SA

Making Changes

Write down what changes you will try or continue in the next couple of weeks.

Changes to carbohydrates
Changes to breakfast
Changes to mealtimes
Any other changes

Attribution-NonCommercial-ShareAlike CC BY-NC-SA

During the next week, please use this to write down diet changes that you try and if you notice any effect.

**What change you made**	**How did you feel when you did this?**

Attribution-NonCommercial-ShareAlike CC BY-NC-SA

Mindfulness Practice: 3 mins coping with difficulty space

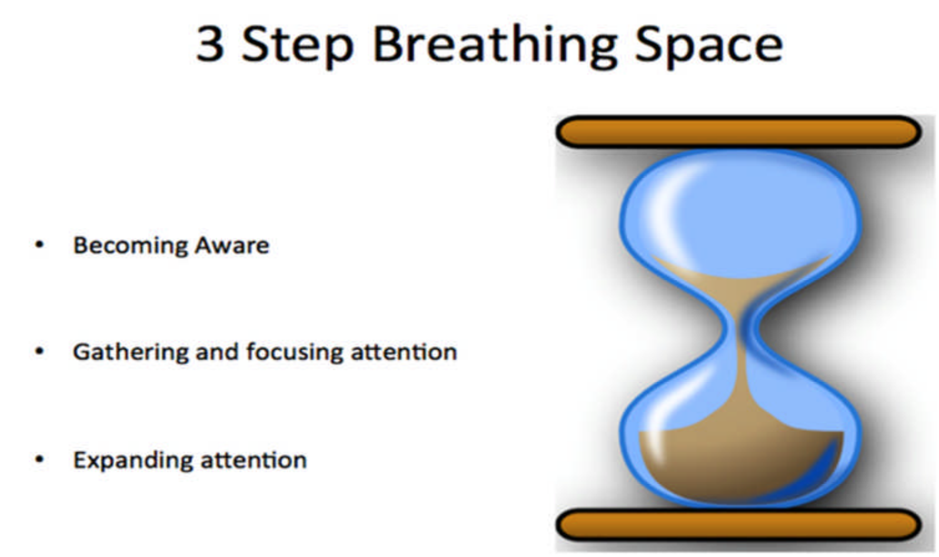

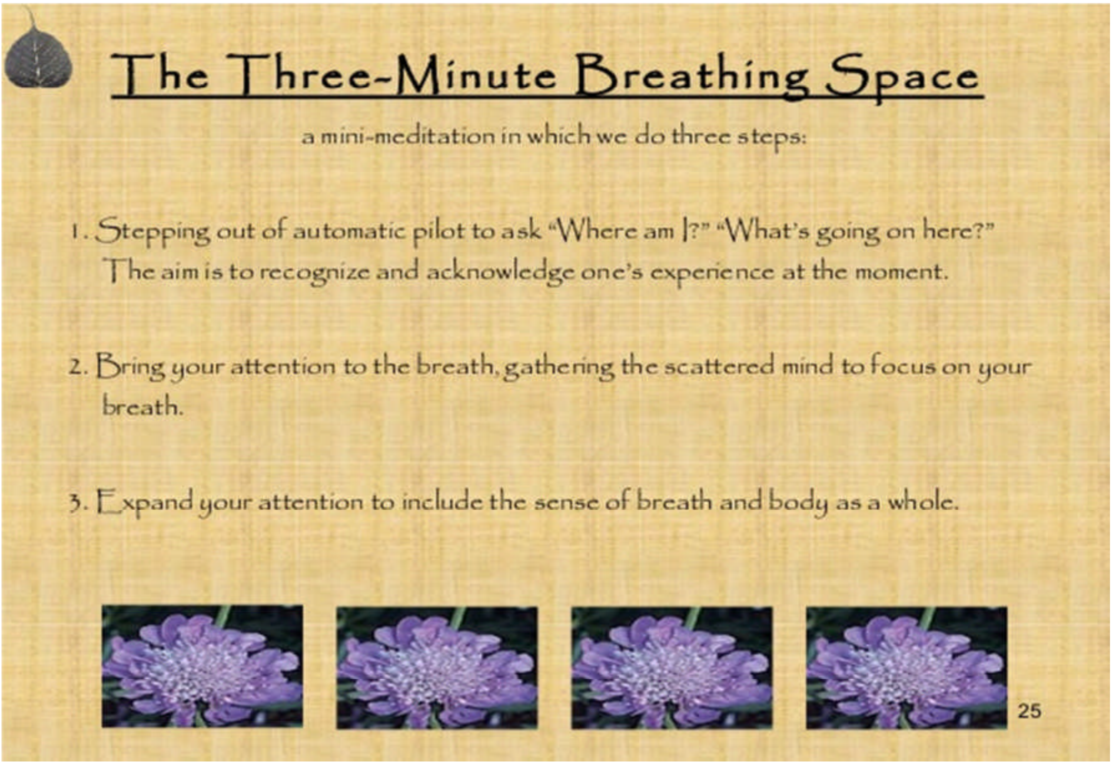

Attribution-NonCommercial-ShareAlike CC BY-NC-SA

Summary

Today, we have talked about:

1. Review of last week
2. Why complex carbohydrates are good for your brain
3. Looking at complex carbohydrates
4. Making social connections
5. Planning what changes you would like to make

FOR NEXT SESSION

Complete the food diary about what changes you have made and if this has made a difference to you

When do you think you might have the opportunity to make these changes?

What might get in the

Attribution-NonCommercial-ShareAlike CC BY-NC-SA

Brain Food

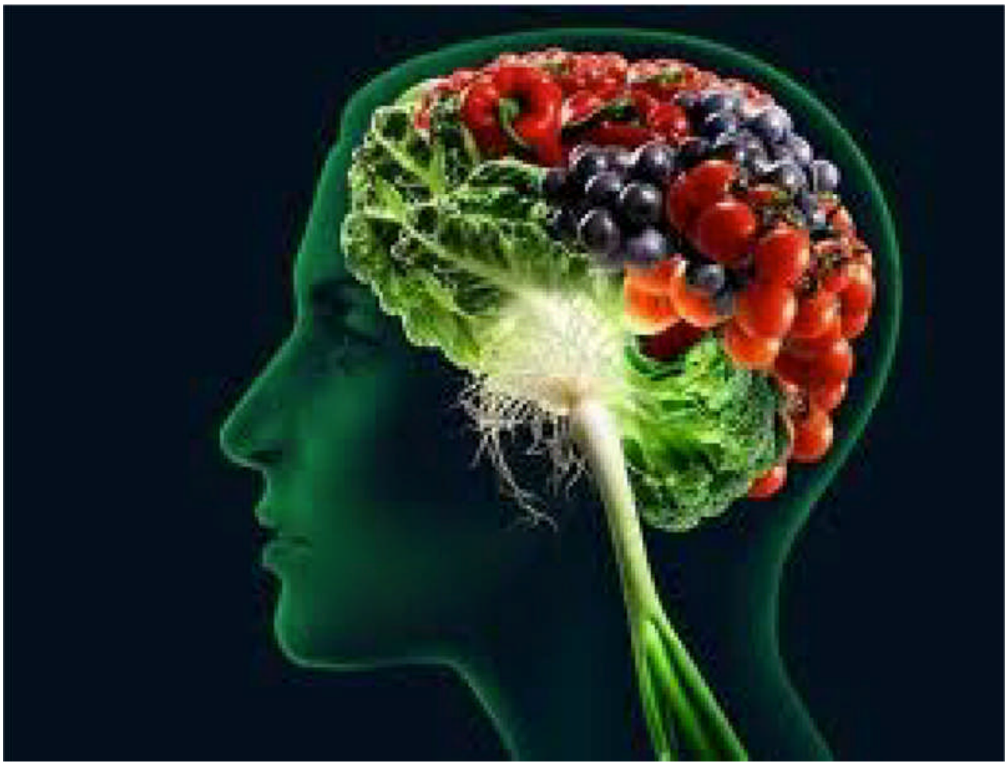

by Dr.Claudia Cooper & Anna Betz
Camden Memory Service
anna.betz@candi.nhs.uk

Session three

Attribution-NonCommercial-ShareAlike CC BY-NC-SA

Session 3: Good and bad fats

Session Plan

- Review of last week
- Looking at fats
    - saturated fats
    - trans fats
    - unsaturated fats
- Why refined oils are bad for you
- Summary and discussion

Attribution-NonCommercial-ShareAlike CC BY-NC-SA

1. What changes did you try last week?

So far we have talked about:

Breakfast

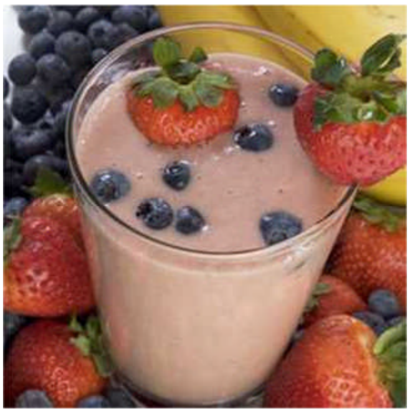

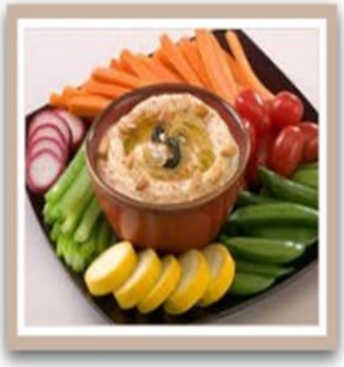

Mealtimes and snacks

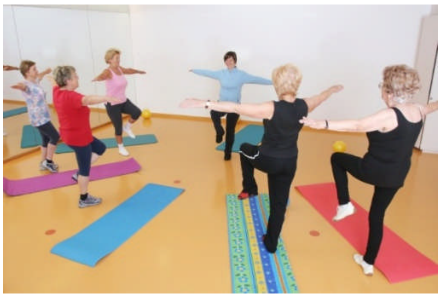

Exercise and social groups

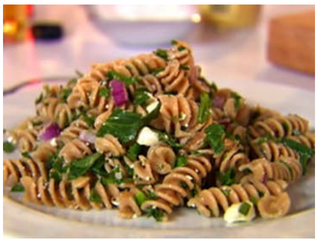

Complex carbohydrates

Attribution-NonCommercial-ShareAlike CC BY-NC-SA

Discussion

What went well?
Were there problems?
What changes will you be continuing?

2. Looking at fats

We all need some healthy fats in our diet, for example to absorb Vitamins A, D, E and K. Certain antioxidants, like lutein in dark green leafy vegetables and lycopene in tomatoes, may also need fat for proper absorption.

There are three different types of fats: **saturated fats, trans-fats, and unsaturated fats.**

We will think about these while sharing some food rich in healthy fats.

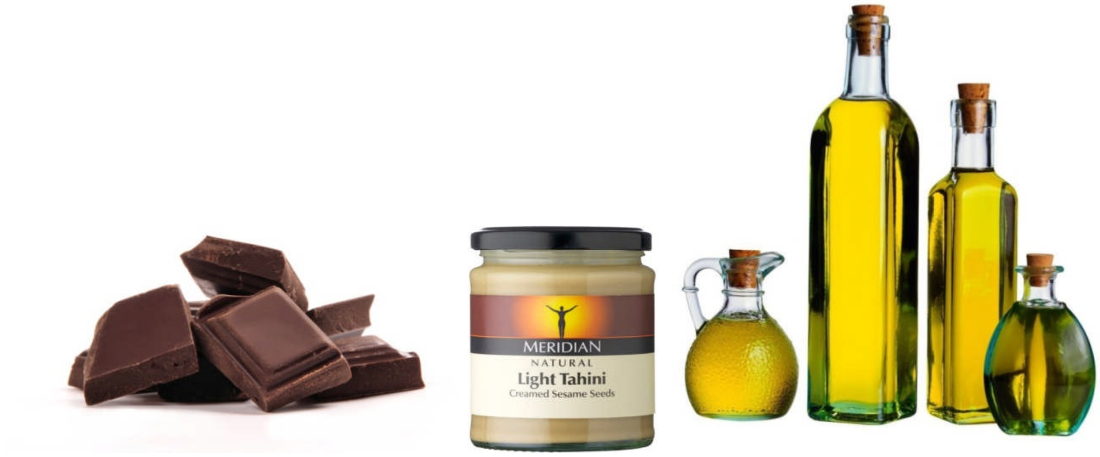

Attribution-NonCommercial-ShareAlike CC BY-NC-SA

Saturated fats

These fats are derived from animal products such as meat, dairy and eggs. They are also found in some plant-based sources such as coconut, palm and palm kernel oils. They raise cholesterol so should only be eaten in small amounts.

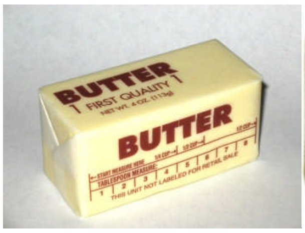

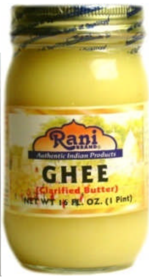

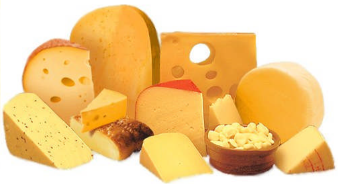

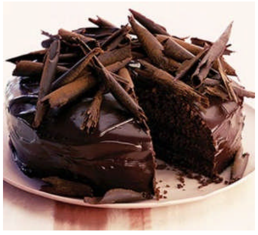
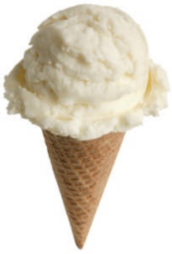
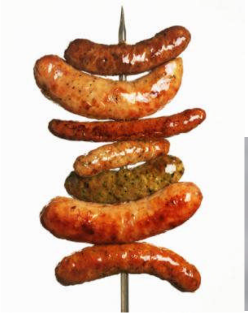
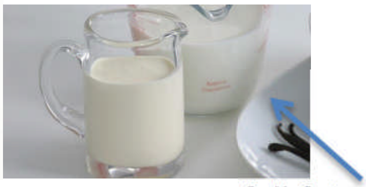

Cream and full fat milk

There is some evidence that saturated fats are not all the same and some in the right quantities might be good for you. Replacing saturated fats with refined carbohydrates increases blood Triglycerides and lowers good (HDL) cholesterol, and these are risk factors for inflammatory diseases including diabetes, dementia and heart disease.

Attribution-NonCommercial-ShareAlike CC BY-NC-SA

Trans-fats

Trans-fats raise the bad cholesterol in your body and lower the good cholesterol that the body needs. They build up in the body and block blood flow to the heart and brain.

Hydrogenation is the chemical process that changes liquid oils into solid fats. Trans-fats, also known as hydrogenated fats, are used for frying or as an ingredient in processed foods. Any item that contains “hydrogenated oil” or “partially hydrogenated oil” probably contains trans-fats.

Transfat is a man-made saturated fat in which a food manufacturer takes an unsaturated fat and treats it with hydrogen atoms to make it into a saturated fat. It is the worst of all fats and the most artery-clogging of all.

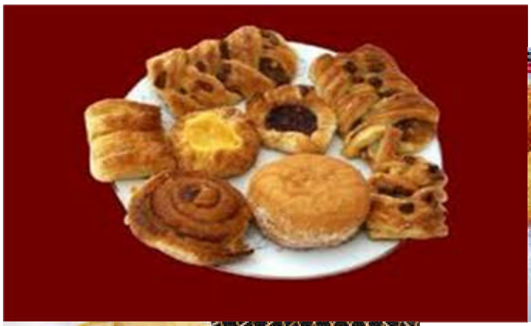
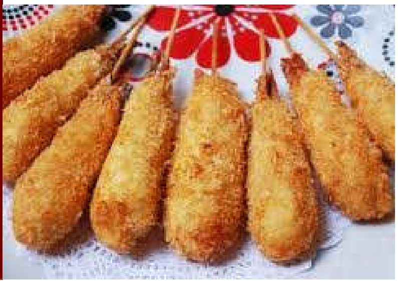
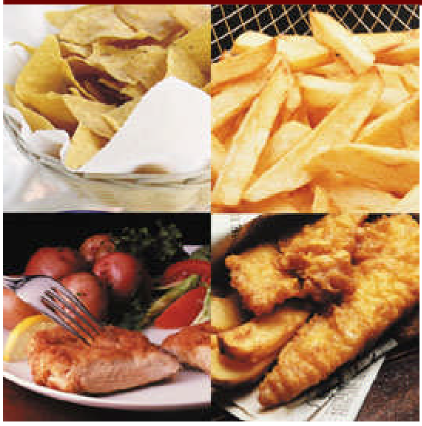

Attribution-NonCommercial-ShareAlike CC BY-NC-SA

Attribution-NonCommercial-ShareAlike CC BY-NC-SA

Unsaturated fats

Regular use of unsaturated fats from whole food sources and cold pressed oils are good for your brain.
Monounsaturated fatty acids (olive, sesame, and avocado oils) are the healthiest for daily use. There are two types:

Monounsaturated fats

They are found in olives, olive oil, nuts, peanut oil and avocados. Some studies have shown that these kinds of fats can lower LDL (bad) cholesterol and maintain HDL (good) cholesterol.

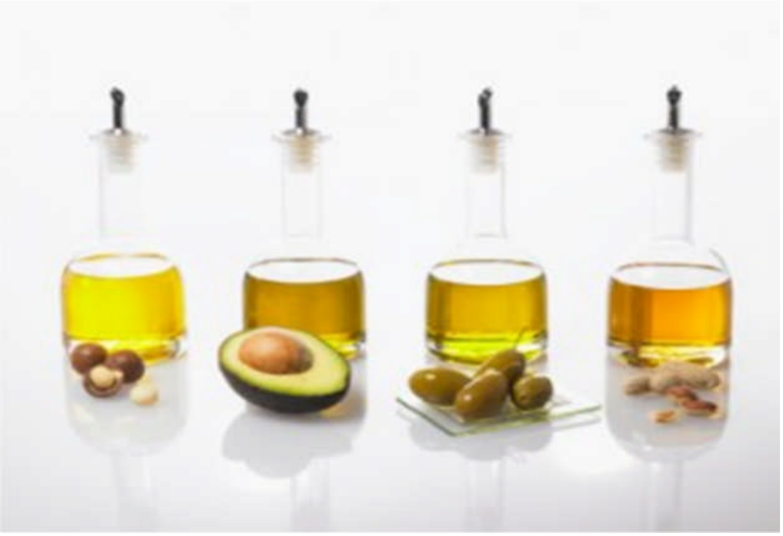

hazelnut avocado olive almond

Myelin, the protective cover around brain nerve fibres, is 70% fat.
One of the most common fatty acids in myelin is **oleic acid**, the main component of olive oil as well as the oils from almonds, pecans, macadamias, peanuts, and avocados.

Attribution-NonCommercial-ShareAlike CC BY-NC-SA

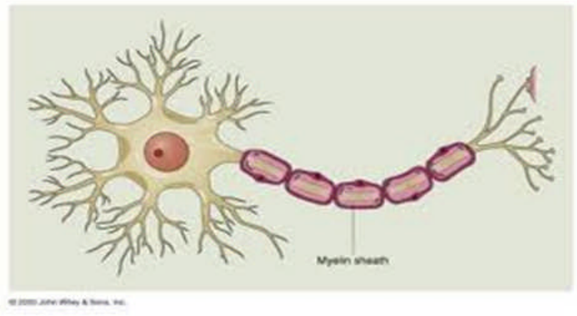

Polyunsaturated fats

These can have a beneficial effect on your health if they are from natural whole food sources and cold pressed oils.

They include **essential fats** that your body needs but can't produce itself – such as **Omega-6** and **Omega-3.**

You must get essential fats through food. Omega-6 and Omega-3 play a crucial role in brain function and overall health.

In the past, people were advised to cut out saturated fats and replace with vegetable oils rich in Omega-6, but current evidence suggests we need to eat Omega 6 and Omega 3 oils in a healthy balance.

Most people consume around 20 times more Omega 6 than Omega-3 and that is bad for the heart and general health.

Omega-6 fats are present in vegetable oils (commonly used for frying in the fast food industry), sunflower, safflower, sesame, corn, rice-bran, soy, peanut, grape seed and wheat oils.

Foods high in omega-3 fats include flax (linseed) oil, deep cold water fish such as sardines, tuna, and wild salmon.

Consuming foods high in Omega 3 oils helps to reduce inflammation. Using too many Omega 6 oils in your diet is bad for you.

Attribution-NonCommercial-ShareAlike CC BY-NC-SA

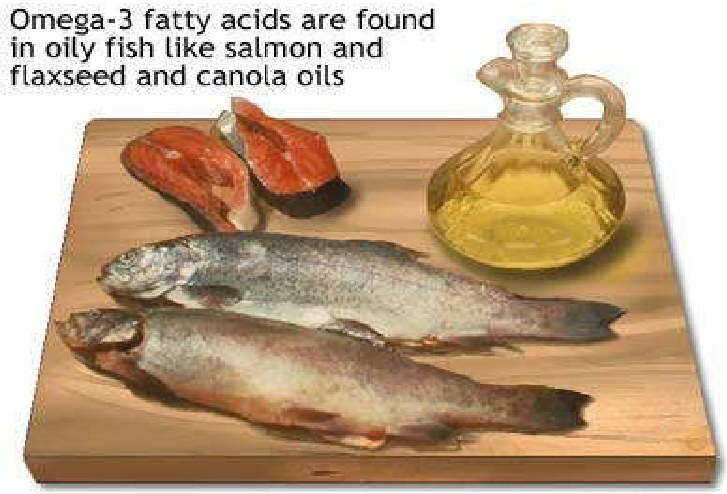

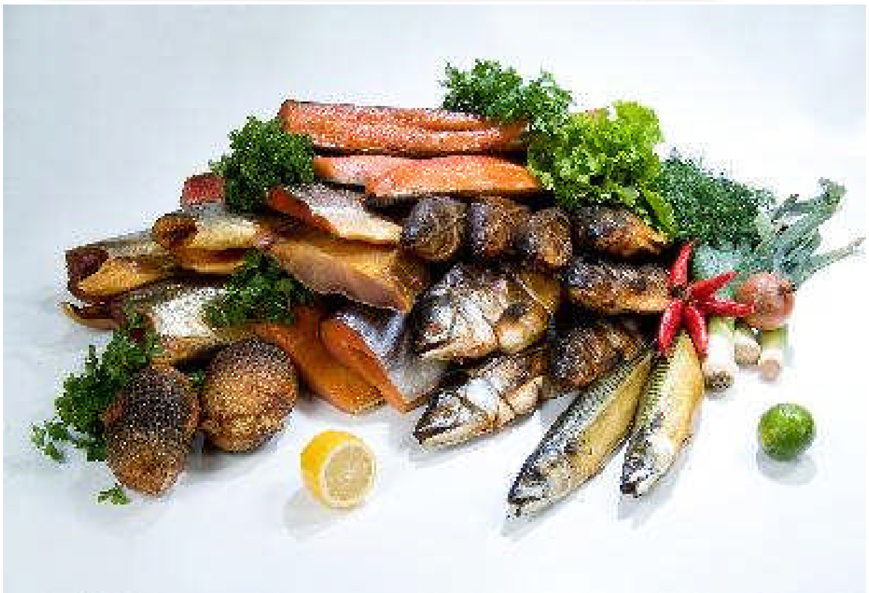

Discussion

What sources of omega-3 do you already eat regularly?

What oils and fats are used to prepare most of your meals?

Attribution-NonCommercial-ShareAlike CC BY-NC-SA

Refined or processed polyunsaturated fats

Polyunsaturated fats are unstable and easily turn into harmful fats if exposed to heat or chemicals used to make refined oils. Manufacturers can heat up unsaturated oils like sunflower seed oil and make it more saturated. Margarine is made in this manner to produce a solid that you can spread.

Polyunsaturated fats in refined products, such as clear vegetable oil, mayonnaise, salad dressings, and most brands of margarine are less healthy than unrefined oils.

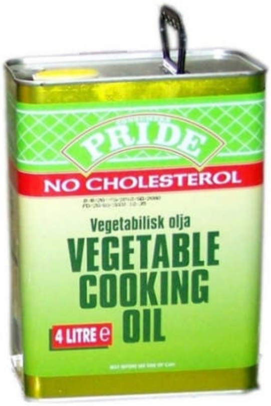

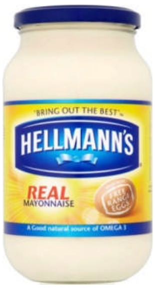

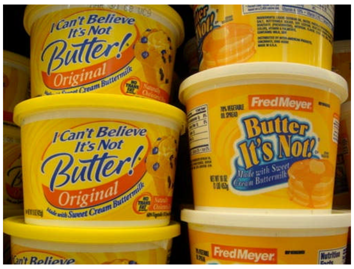

Attribution-NonCommercial-ShareAlike CC BY-NC-SA

Cooking oils

When heating any oil, it is important to keep them below their smoke point, (before oil burns to the point of smoking).

Oils heated above their stability point begin to decompose, releasing free radicals along with toxic fumes.

The BBC investigated the "healthiest" oils to cook with for its series Trust Me I'm A Doctor in 2015.

Volunteers were given sunflower oil, vegetable oil, corn oil, cold-pressed rapeseed oil, olive oil, butter, goose fat and lard to use in cooking.

Cooking with sunflower oil and corn oil produced aldehydes, which can cause cancer at levels 20 times higher than recommended by the World Health Organisation. Olive oil and rapeseed oil produced far fewer aldehydes as did butter and goose fat.

Presenter Michael Mosley said: "Put simply, cooking with these oils is producing even more toxic compounds than has ever before been realised. In contrast, the olive oil and cold-pressed rapeseed oil produced far fewer aldehydes, as did butter and goose fat. The reason being that these fats are richer in monounsatured and saturated fats, and are much more stable when heated.

Attribution-NonCommercial-ShareAlike CC BY-NC-SA

Here are some unrefined oils you could use in preparing meals and in cooking:

Cooking Temperature	Oils	Cooking Uses
No Heat or low Heat	Flaxseed (high in omega-3); Hempseed oil; Sunflower	Salad, dressings, steam, simmer
Up to Medium-Low Heat	Coconut, Olive, Sesame,	Low heat baking, light sauté, pressure-cook
Up to Medium-High Heat	Macadamia nut, Olive oil, Coconut oil, Cold pressed Rapeseed oil, Butter, Goosefat, Ghee	Medium-heat baking, sauté, stir fry, wok
Up to High Heat	Avocado,	Brown, deep-fry, sear

Attribution-NonCommercial-ShareAlike CC BY-NC-SA

Every day we recommend:

2+ vegetable servings. Raw vegetables are particularly good for you.

3+ fruit portions (including natural fruit juices)

4+ tablespoons of olive oil

Every week:

3+ servings of legumes a week

2+ servings of Sofrito (sauce made with tomato and onion, leek or garlic and simmered with olive oil)

3+ servings of fish (especially fatty fish), seafood

But try to avoid:

Sugary drinks
Commercial bakery goods, sweets, and pastries
Spread fats
Red and processed meats (have <1 serving/day)

Attribution-NonCommercial-ShareAlike CC BY-NC-SA

Making changes

How could you introduce good fats into your diet ?

Write down what changes you will try, or keep trying in the next couple of weeks.

Changes to breakfast<br><br><br><br>Changes to lunch<br><br><br><br>Any other evening meal<br><br><br><br>Changes to snacks

Attribution-NonCommercial-ShareAlike CC BY-NC-SA

During the next week, please use this to write down diet changes that you try and if you notice any effect.

What change you made	How did you feel when you did this?

BY NC SA
Attribution-NonCommercial-ShareAlike CC BY-NC-SA

Mindfulness Practice: 3 mins coping with difficulty space

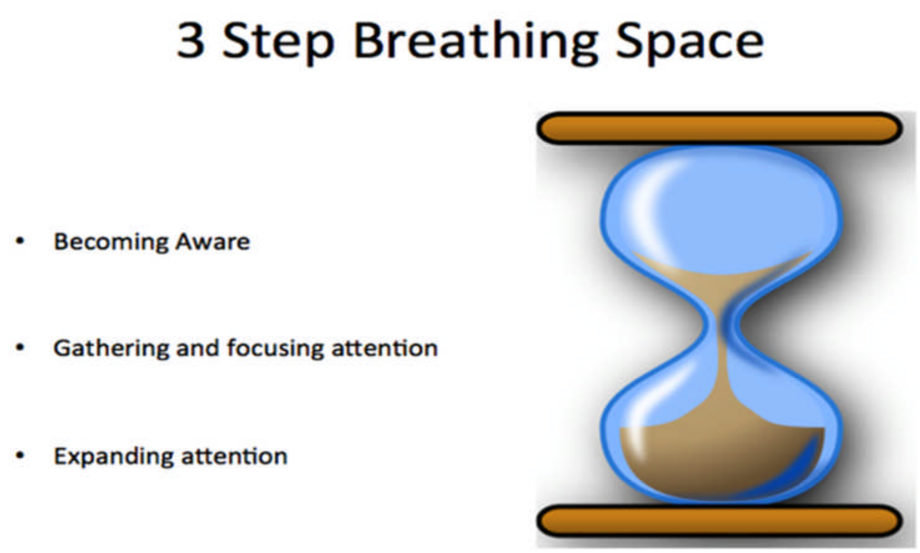

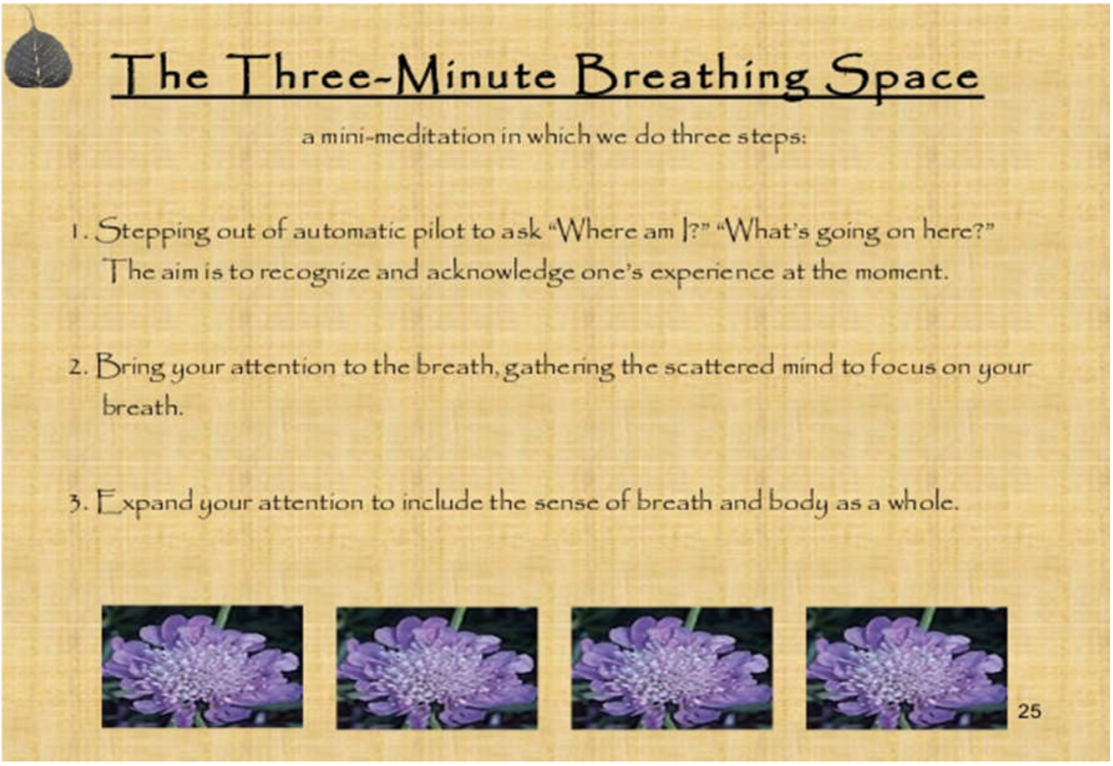

Attribution-NonCommercial-ShareAlike CC BY-NC-SA

FOR NEXT SESSION

Complete the food diary about what changes you have made and if this has made a difference to you

When do you think you might have the opportunity to make these changes?

What might get in the way?

Summary

Today, we have talked about:

Types of fats

- saturated fats
- trans fats
- unsaturated fats

Trying to use unrefined oils

Attribution-NonCommercial-ShareAlike CC BY-NC-SA

Brain Food

by

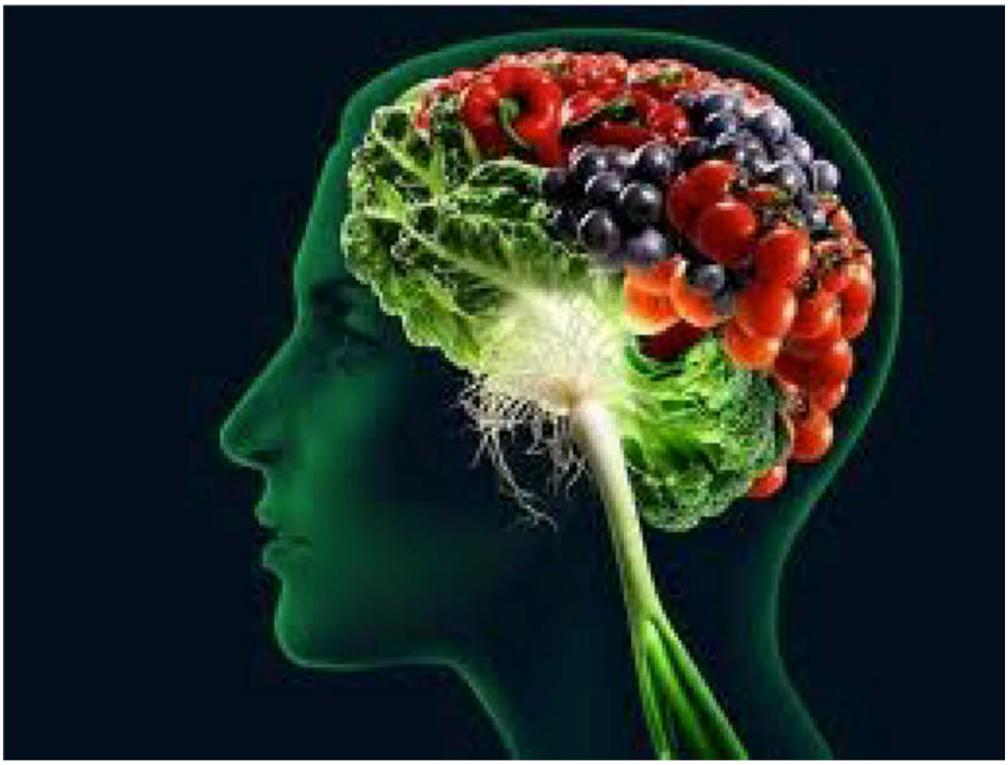

Dr.Claudia Cooper & Anna Betz
Camden Memory Service
anna.betz@candi.nhs.uk

Session four

Attribution-NonCommercial-ShareAlike CC BY-NC-SA

Session 4: Mediterranean diet

Session Plan

- Review of last week
- Looking at Mediterranean diet
- Planning changes
- Summary and discussion

Attribution-NonCommercial-ShareAlike CC BY-NC-SA

1. What changes have you tried?

So far we have talked about:

Breakfast

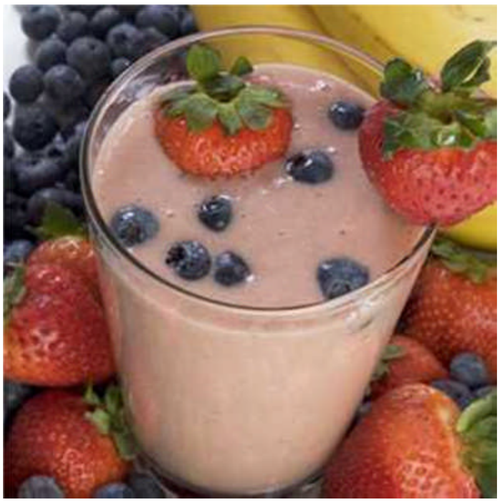

Complex carbohydrates

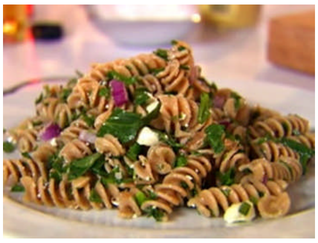

Monounsaturated fats and unrefined polyunsaturated fats

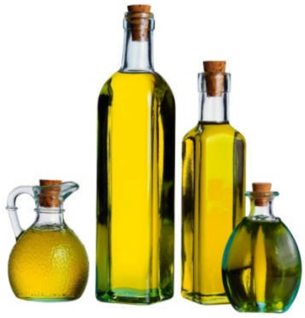

What went well?
Were there problems?
What changes will you be continuing?

Attribution-NonCommercial-ShareAlike CC BY-NC-SA

2. Looking at the Mediterranean diet

From salads of tomato, cucumber and feta to moussaka and tzatziki, the Mediterranean diet's mix of whole grains, fresh vegetables and oily fish has proven incredibly healthy and good for the brain.

The Mediterranean diet has been associated with:

- A lower risk of diabetes and obesity
- a lower risk of heart disease, high blood pressure and stroke
- a reduced risk of Alzheimer's disease

It is a diet that brings together many of the diet changes we have discussed so far.

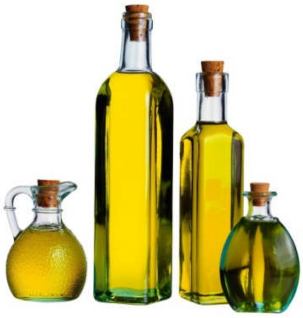

Attribution-NonCommercial-ShareAlike CC BY-NC-SA

Mediterranean Diet Pyramid

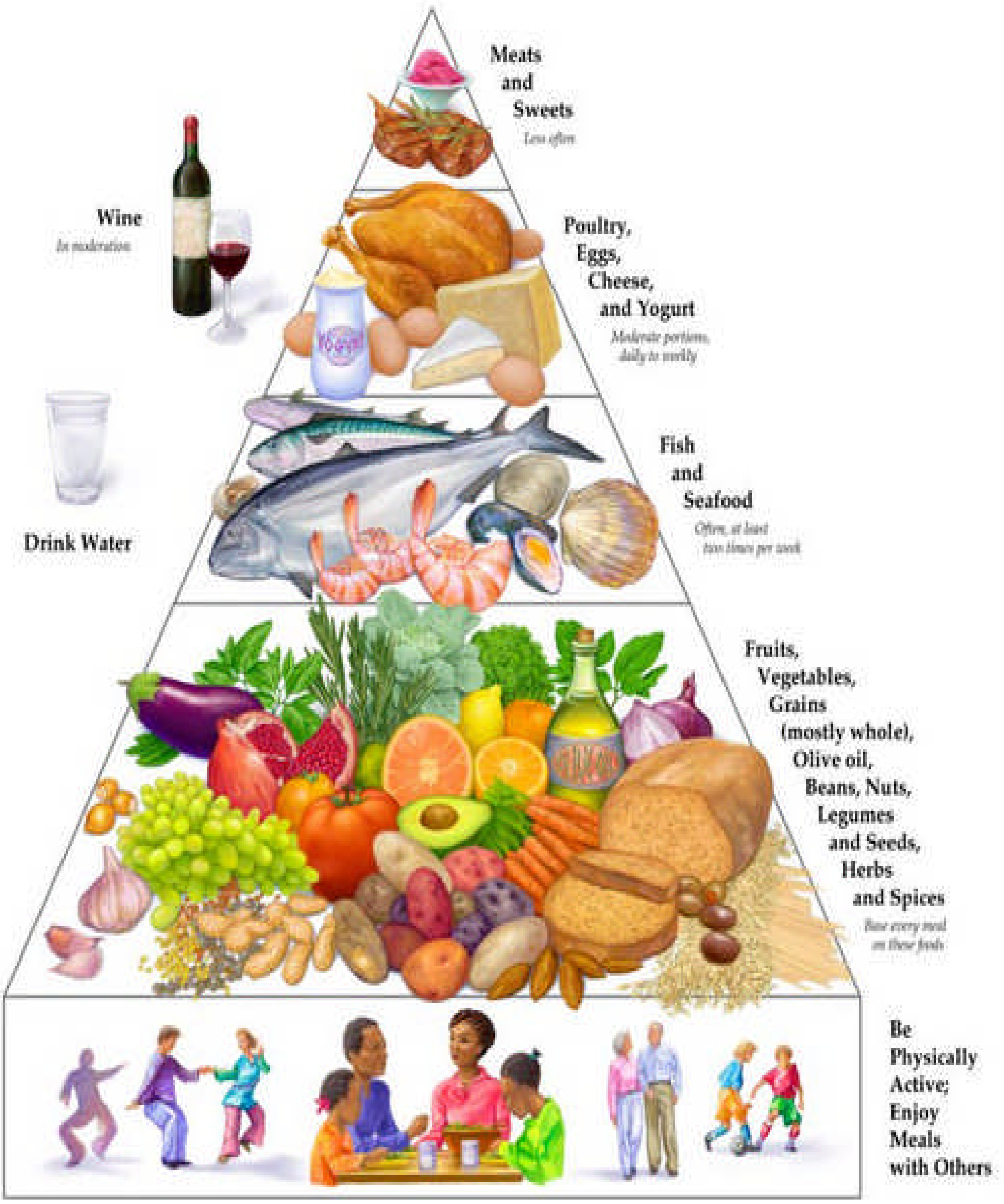

Attribution-NonCommercial-ShareAlike CC BY-NC-SA

Key Concepts of Mediterranean diet

- Plenty of virgin olive oil for cooking and dressing of dishes
- 2+ servings (125g/serving) per day of vegetables
- 3+ servings per day of fresh fruits (including natural juices);
- 3+ servings (40 g/serving) per week of legumes;
- 3+ servings (150 g/serving) per week of fish or seafood
- 3+ servings (25 g/serving) per week of nuts or seeds;
- Limit red meats or processed meats (ie, burgers and
- Sausages)
- Regularly cooking (at least twice a week) with salsa made with minced tomato, garlic, and onion simmered in olive oil, adding other aromatic herbs or not, for dressing different dishes.
- Limit dairy products, sweet foods and processed foods.
- Drink (red) wine during meals, but no more than 1-2 small glasses per day.
- Water is the best 'non-alcoholic beverage' (as opposed to sugary drinks), although health benefits have also been claimed for various teas and coffee. You can make water taste more refreshing by adding juice of fresh lemon.

Attribution-NonCommercial-ShareAlike CC BY-NC-SA

Fish

The Mediterranean diet includes plenty of fish. A mix of oily and white fish (and shell fish) in the diet is a good source of protein that reduces the risk of heart disease.

Oily fish (and some shellfish) contain cardio-protective omega 3 fatty acids, vitamins A and D. Whole fish are a source of calcium and phosphorus.

If you have high total cholesterol levels, avoid eating too much shrimp.

White fish

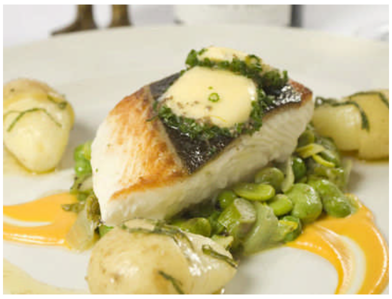

Examples are cod, haddock, plaice, pollack, coley, dab, flounder, red mullet, gurnard and tilapia. They are very low in fat, and contain some omega-3 fatty acids, but much less than oily fish

Attribution-NonCommercial-ShareAlike CC BY-NC-SA

Oily fish

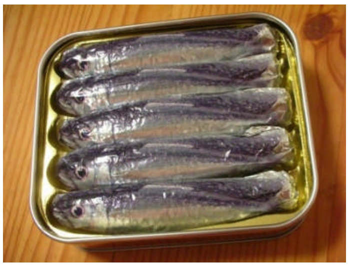 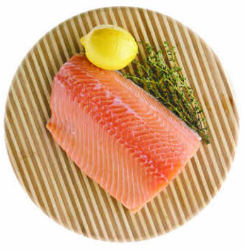

These are a rich source of omega 3. They include: sardines, salmon, anchovies, carp, herring, mackerel, pilchards, trout, tuna (fresh) and whitebait.
Canned tuna does not count as oily fish. Fresh tuna is an oily fish, but when it is canned the amount of omega-3 fatty acids is reduced to levels similar to those in other fish.

Shellfish

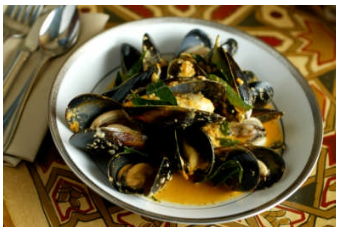 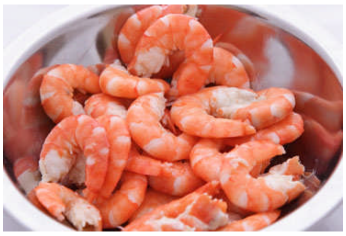

These include: prawns, mussels and langoustine. They are low in fat and a good source of selenium, zinc, iodine and copper.
Some types, such as mussels, oysters, squid and crab are also good sources of long-chain omega-3 fatty acids, but they do not contain as much as oily fish.

Attribution-NonCommercial-ShareAlike CC BY-NC-SA

What’s wrong with too much red meat?

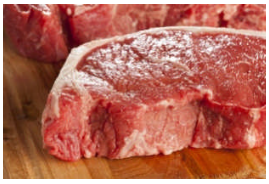 Red meat is a good source of protein and vitamins and minerals, such as iron and zinc.

Eating too much red and processed meat increases the risk of bowel and other cancers, diabetes, obesity, hypertension and arthritis. Processed meat increases the risk of cardiovascular disease.

National guidelines suggest you don’t eat more than three thin-cut slices of roast beef, lamb or pork a week.

Why limit dairy foods ?

These are a good source of calcium and vitamins, but most of the fat they contain is saturated fat. Cheese can also be high in salt.

Lactose, the sugar in milk is a simple sugar composed of glucose and galactose. This composition results in lactose’s ability to produce insulin spikes.

Milk from grass fed cows contains some Omega-3 fatty acids. Research shows that cows feeding exclusively on green grass contains more omega-3 fatty acids than milk from cows fed conserved grass.

Insoluble fiber, such as that found in wheat bran, reduces calcium absorption; but soluble fiber, such as that found in psyllium and fruit pectins, does not seem to affect absorption.

BY NC SA

Attribution-NonCommercial-ShareAlike CC BY-NC-SA

Brain-healthy recipes

Most of your favourite foods can be prepared so they are **brain-healthy.** For the few foods that cant' be made more brain-healthy, enjoy them in moderation.

<u>Brain-healthier Pepperoni Pizza</u>

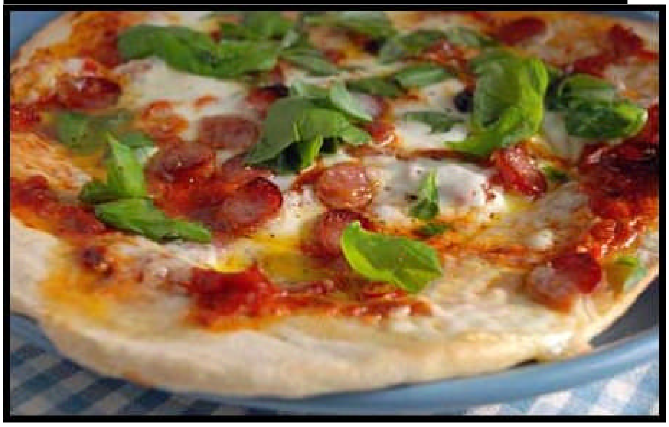

- 1 wholemeal flat bread
- 12 slices of turkey with pepperoni
- 4oz tomato sauce
- 4oz fat free mozzarella cheese
- 1oz mix of oregano, black pepper, crushed red pepper and parsley or fresh basil

<u>Tasty brain-healthy salad dressing</u> can be made with extra virgin olive oil, hempseed oil, flaxseed oil, or hazelnut oil and apple cider vinegar or fresh lemon as well as fresh or dried herbs and a little mustard.

Attribution-NonCommercial-ShareAlike CC BY-NC-SA

Greek Salad with Chicken

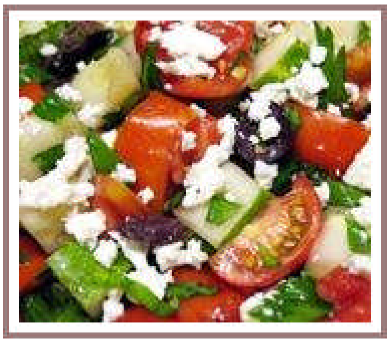

one lettuce, 3 oz chicken, 2 sliced tomatoes, 4oz sliced onion, half a cucumber, slice, ½ oz feta cheese, 2 olives, 1 teaspoon olive oil, 1 tablespoon cider vinegar or lemon

Mix lettuce, tomatoes, onions, cucumber and olives in a bowl. Top with chicken, cheese and oil and vinegar/lemon dressing.

Tuna Salad

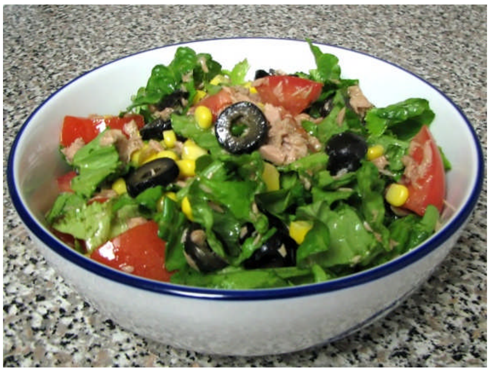

3 oz tuna, 2 tablespoons chopped red onion, 2 tablespoons chopped celery, half a cucumber (chopped), 4oz chopped red peppers, one lettuce, 1 apple, 4 oz low fat yogurt.

Dressing: 1 tablespoon light mayonnaise, 1 teaspoon olive oil, 1 tablespoon cider vinegar or lemon. (optional)

Mix dressing ingredients together. Mix tuna, onion, celery, cucumber and peppers. Add dressing and serve on a bed of lettuce or spinach.

Attribution-NonCommercial-ShareAlike CC BY-NC-SA

How much?

Every day we recommend:

2+ vegetable servings. Raw vegetables are particularly good for you.

3+ fruit portions (including natural fruit juices)

4+ tablespoons of olive oil

Every week:

3+ servings of legumes a week

2+ servings of Sofrito (sauce made with tomato and onion, leek or garlic and simmered with olive oil)

3+ servings of fish (especially fatty fish), seafood

But try to avoid:

Sugary drinks
Commercial bakery goods, sweets, and pastries
Spread fats
Red and processed meats (have <1 serving/day)

Attribution-NonCommercial-ShareAlike CC BY-NC-SA

Mindfulness Practice: 3 mins coping with difficulty space

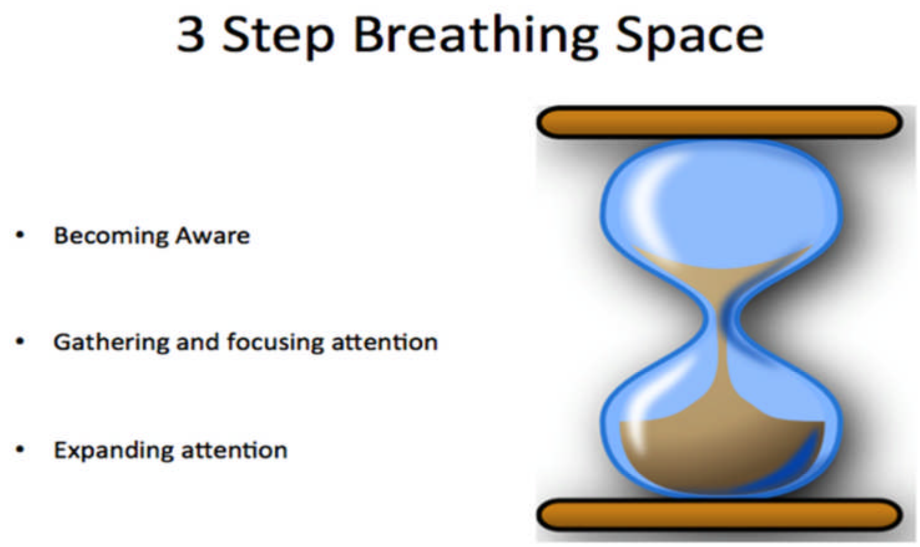

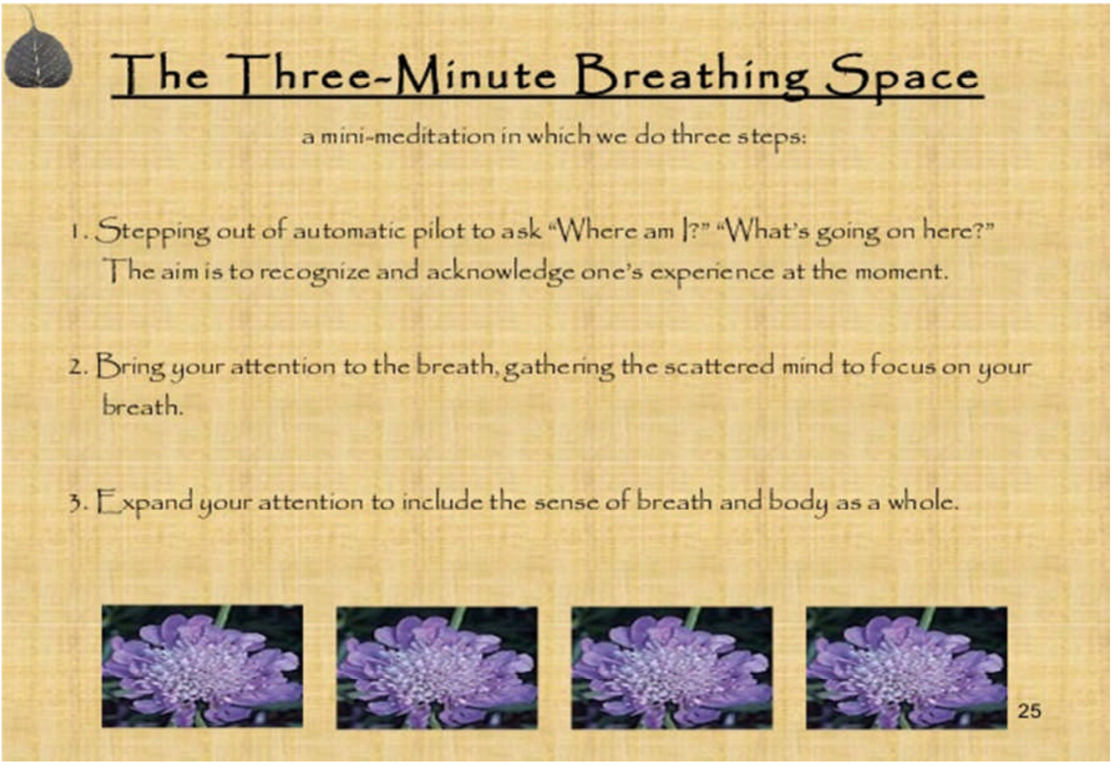

Attribution-NonCommercial-ShareAlike CC BY-NC-SA

3. Planning changes

During the next week, please use this to write down diet changes that you try and if you notice any effect.

What change you made	How did you feel when you did this?

Summary

Today, we have talked about:

Mediterranean diet

FOR NEXT SESSION

Complete the food diary about what changes you have made and if this has made a difference to you

When do you think you might have the opportunity to make these changes?

What might get in the way?

Attribution-NonCommercial-ShareAlike CC BY-NC-SA

Brain Food

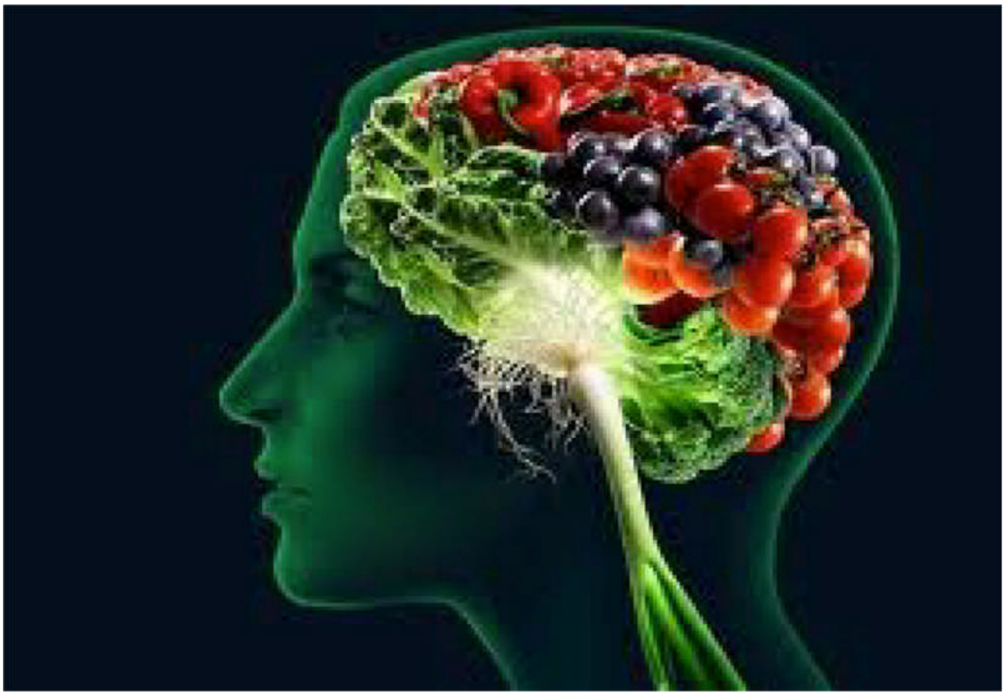

by Dr.Claudia Cooper & Anna Betz
Camden Memory Service
anna.betz@candi.nhs.uk

Session five

Attribution-NonCommercial-ShareAlike CC BY-NC-SA

Session 5: Vitamins, Minerals and putting it all together

Session Plan

- Review of last week
- Vitamins and anti-oxidants
- Drinks
- Review of the course
- Summary and discussion

Attribution-NonCommercial-ShareAlike CC BY-NC-SA

What changes have you tried?

So far we have talked about:

Breakfast

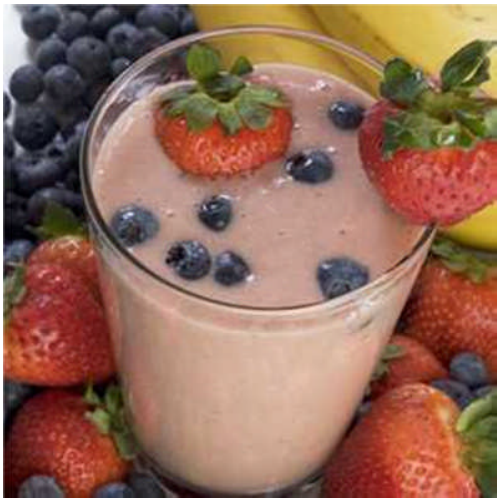

Complex carbohydrates

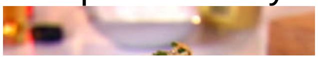

Mediterranean diet

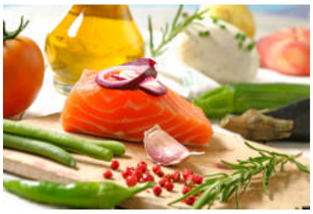

Monounsaturated fats and
unrefined polyunsaturated fats

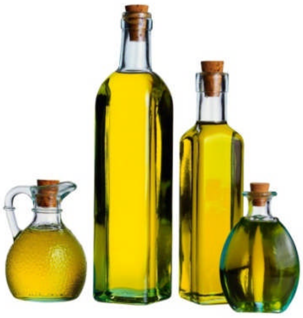

What went well?
Were there problems?
What changes will you be continuing?

Attribution-NonCommercial-ShareAlike CC BY-NC-SA

Vitamins and anti-oxidants

A brain-healthy diet will help increase your intake of vitamins and the trace elements necessary for the body to use them effectively.

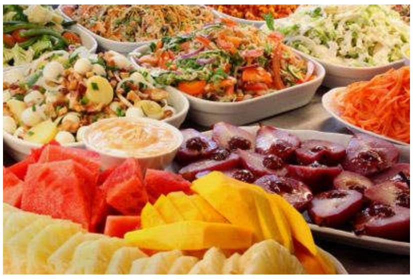

The B vitamins, especially thiamine (vitamin B1), niacin (Vitamin B3), folate (folic acid), vitamin B6 and vitamin B12 are needed to metabolise glucose, the brain's primary fuel.

They also help regulate blood levels of homocysteine, an amino acid made when the body breaks down protein. High levels of homocysteine are associated with heart disease.

Supplements can help people who do not get enough B vitamins from their diet.

B vitamins are found in:

Thiamine	Niacin	Folate	B6	B12
vegetables peas, fresh and dried fruit<br><br>wholegrain breads, some fortified cereals<br><br>eggs<br><br>liver	wheat flour maize flour<br><br>Eggs, milk<br><br>meat<br><br>fish	Broccoli, brussels sprouts spinach, asparagus, peas<br><br>chickpeas, brown rice fortified cereals<br><br>liver	Vegetables potatoes<br><br>Bread, cereals, such as oatmeal, wheat germ and rice, fortified cereals<br><br>Soya beans, peanuts<br><br>Eggs, milk<br><br>Pork, poultry, tuna	some fortified breakfast cereals<br><br>Eggs, milk, cheese<br><br>Meat, salmon,cod

Attribution-NonCommercial-ShareAlike CC BY-NC-SA

Antioxidants

Certain nutrients, such as vitamins E and C, are antioxidants. Oxidative damage to brain cells occurs in Alzheimer's disease, and antioxidants might help prevent this, although this hasn't been proven yet.

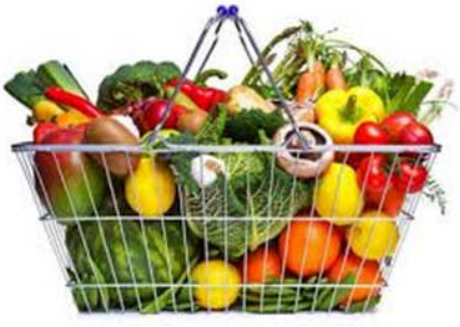

Dark-skinned fruits and vegetables have the highest levels of antioxidants. Such vegetables include: kale, spinach, cabbage, Brussels sprouts, alfalfa sprouts, broccoli, beets, red bell pepper, onion, corn and aubergine, artichokes and other green vegetables.

Fruits with high antioxidant levels include prunes, raisins, blueberries, blackberries, strawberries, raspberries, plums, red grapes and cherries.

Other foods high in flavonoids that also may provide some benefit: Tea, dark chocolate, citrus fruits, red wine.

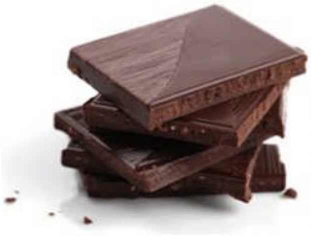

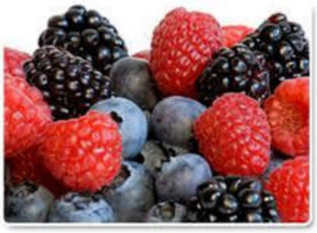

Attribution-NonCommercial-ShareAlike CC BY-NC-SA

Other antioxidants and plant compounds such as **Coenzyme Q10, Ginkgo biloba and Curcumin** (in turmeric) are also being studied for potential benefits in brain health.

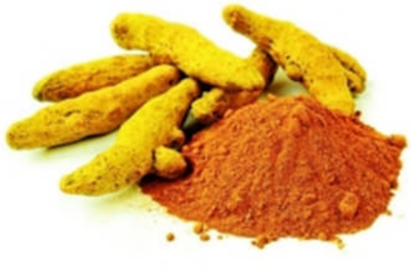

Nuts

Nuts are packed with protein, fibre and essential fats.

They are high in fat, but much of it is heart-healthy. The amounts of saturated fat varies between nuts. Brazil nuts, Macadamias and Cashews have the highest saturated fat content.

They contain lots of vitamins and minerals:

	HEALTH BENEFITS
Almonds	High in vitamin E
Walnuts	Antioxidants, omega 3,
Cashews	Iron, zinc, magnesium
Hazelnuts	Folic acid
Pecans	Antioxidants, B3, oleic acid
Pistachios	B6, potassium

Attribution-NonCommercial-ShareAlike CC BY-NC-SA

Herbs and Spices

Herbs and spices are also antioxidants. The higher the **ORAC** (Oxygen Radical Absorbance Capacity) score the more powerful an antioxidant a substance is.

	ORAC SCORE	OTHER PROPERTIES/ACTIONS
Cloves	290,283	Strongly antibacterial & antifungal
Oregano	175,295	Strongly antifungal
Rosemary	165,280	Helps digestion, stimulates circulation
Thyme	157,380	Relaxes lungs, antibacterial
Cinnamon	131,420	Regulates blood sugar, antibacterial
Turmeric	127,068	Improves digestion, reduces cholesterol, anti-inflammatory
Parsley	74,000	Kidney tonic, diuretic, Vit C, Iron, Vit A
Marjoram	27,297	Minerals, Vit A,C,K, helps sleep & digestion

Drinks

Caffeine/coffee

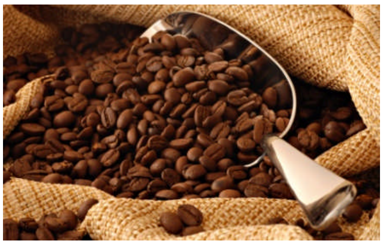

Drinking 1-2 cups of coffee earlier in the day might help memory – people who drank coffee in mid life were less likely to get dementia in one study, and animal studies also suggest it might be helpful.

Drinking coffee after lunch is not recommended, as it may affect the sleep-wake cycle and impair the ability to effectively fall asleep (even up to 10 hours after drinking it!)

Caffeine may also have detrimental effects on the heart such as increasing the heart rate and may increase anxiety.

Attribution-NonCommercial-ShareAlike CC BY-NC-SA

Alcohol

There is no evidence that alcohol helps memory and too much can certainly make it worse.

1 glass of red wine with a meal is considered helpful due to the flavonoid content.

Consumption of more than 1 glass can lead to negative health consequences.

Beer and wine vary in their sugar content but be aware that all beers and wines contain some carbohydrates and more calories than most people expect.

Fruit Juice

In general it is preferable to eat whole fruit rather than drink fruit juice due to its high sugar content. Try and dilute it with carbonated water for a refreshing drink.

Tea

2-3 cups of tea can help brain health thanks to compounds called polyphenols also present in green tea.

Coca-cola and other soft drinks

There are 65 g of sugar in a typical 20oz bottle of cola. That's about 15 teaspoons of sugar! Remember sugar is a bad carbohydrate. Soft drinks are high in sugar.

Try to avoid sugared beverages, including those sweetened by high-fructose corn syrup (same thing as sugar).

Beware of products labelled "juice drink" or "juice cocktail". These are not juice. Most contain a little fruit juice (15%) and the rest is sugar or high fructose corn syrup.

Attribution-NonCommercial-ShareAlike CC BY-NC-SA

Beer and wine vary in their sugar and caloric content but be aware that all beers and wines contain some carbohydrates and more calories than most people expect.

Lemon water

Fresh lemon juice squeezed into a glass of hot or cold water is a natural energiser. It makes you feel revitalised and refreshed.

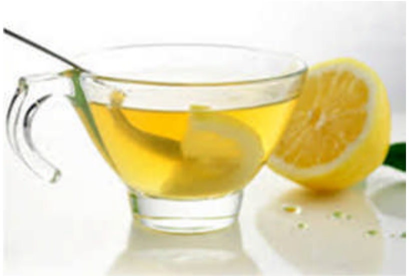

How much?

Every day we recommend:

2+ vegetable servings. Raw vegetables are particularly good for you.

3+ fruit portions (including natural fruit juices)

4+ tablespoons of olive oil

Every week:

3+ servings of legumes a week

Attribution-NonCommercial-ShareAlike CC BY-NC-SA

2+ servings of Sofrito (sauce made with tomato and onion, leek or garlic and simmered with olive oil)

3+ servings of fish (especially fatty fish), seafood

3+ servings (30g) of nuts (e.g. walnuts, hazelnuts, almonds)

But try to avoid:

Sugary drinks
Commercial bakery goods, sweets, and pastries
Spread fats
Red and processed meats (have <1 serving/day)

Write down what changes you have made which you would like to carry on with

**What change you made**

Attribution-NonCommercial-ShareAlike CC BY-NC-SA

Summary

Today, we have talked about:

Vitamins and Minerals
Drinks
We revised the course
Planned changes to continue with

Thank you for coming to our course. We hope you have enjoyed it and found it helpful.

Attribution-NonCommercial-ShareAlike CC BY-NC-SA

Mindfulness Practice: 3 mins coping with difficulty space

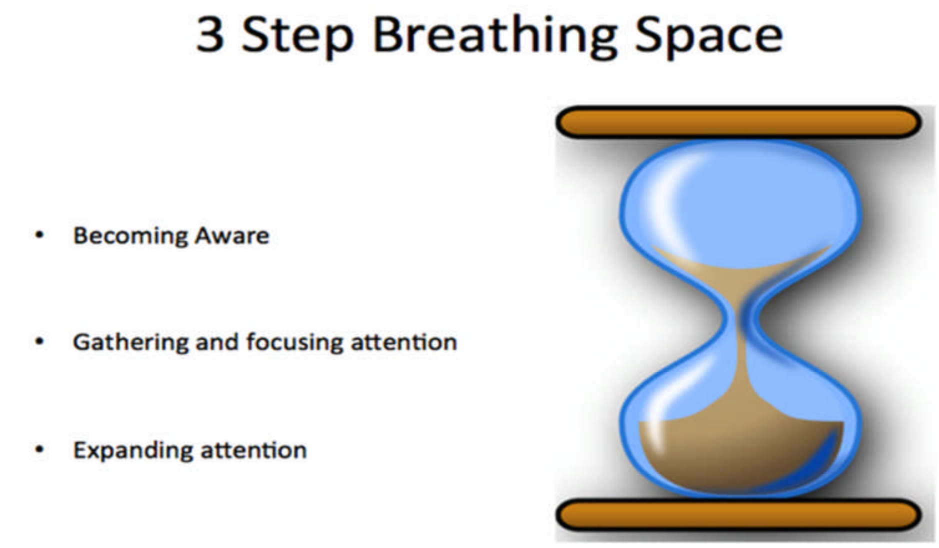

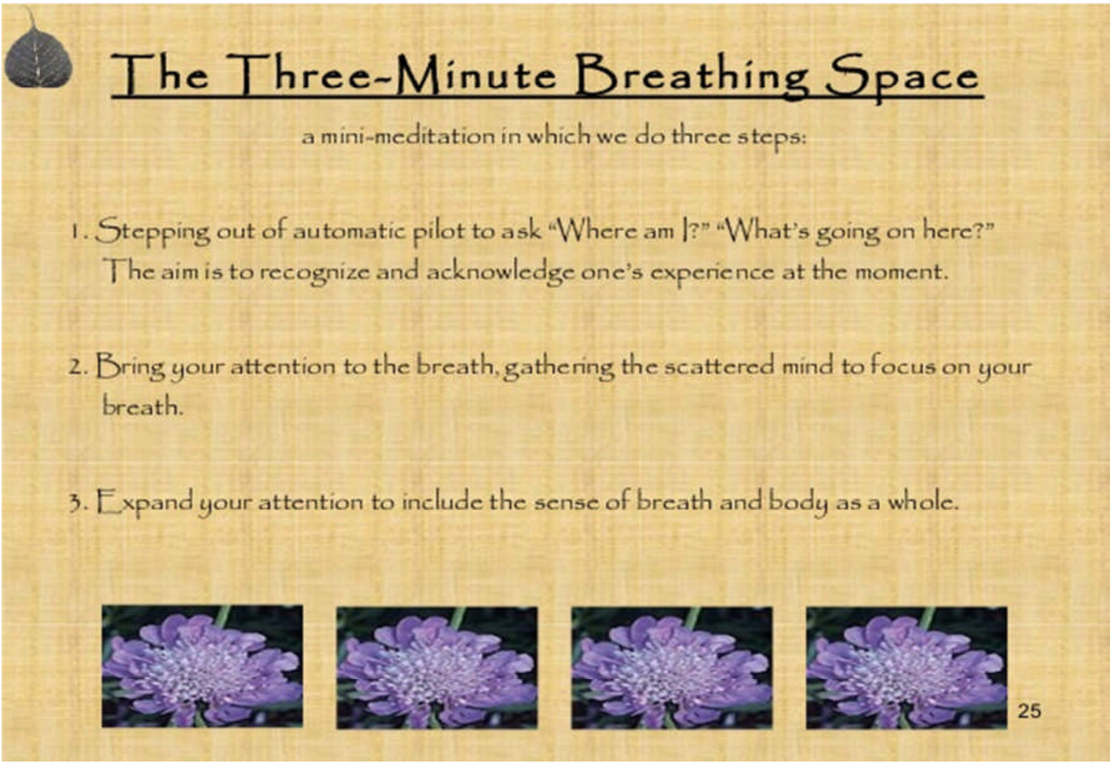

Attribution-NonCommercial-ShareAlike CC BY-NC-SA

Brain food

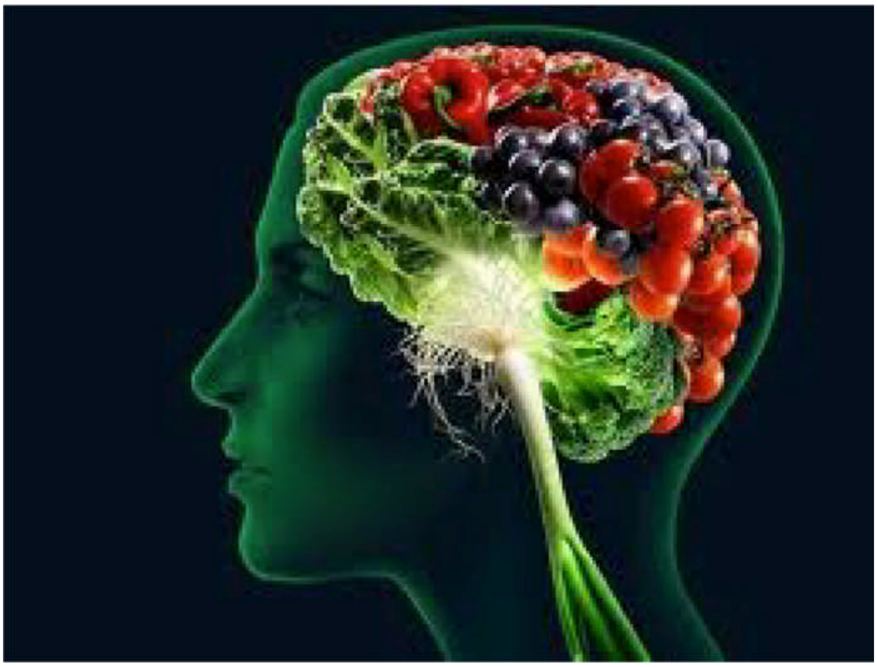

by Dr.Claudia Cooper & Anna Betz
Camden Memory Service
anna.betz@candi.nhs.uk

Reunion

Attribution-NonCommercial-ShareAlike CC BY-NC-SA

Looking back at the five sessions

Session 1: Starting well

Session 2: Good and bad carbohydrates

Session 3: Good and bad fats

Session 4: Mediterranean diet

Session 5: Vitamins, minerals and putting it all together

Attribution-NonCommercial-ShareAlike CC BY-NC-SA

Session 1: Starting well

- We talked about the importance of a good breakfast for a good start to the day.
- Oats or oatmeal make a good breakfast because energy is released slowly.
- Its good to have some protein to keep your energy levels up, for example
  - pumpkin seeds, walnuts, pecan or other nuts on cereal.

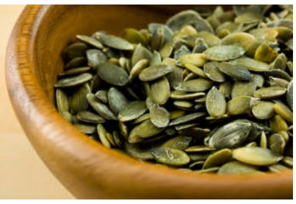

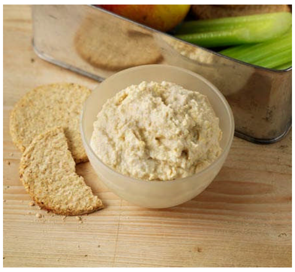

  - Salmon with tomatoes

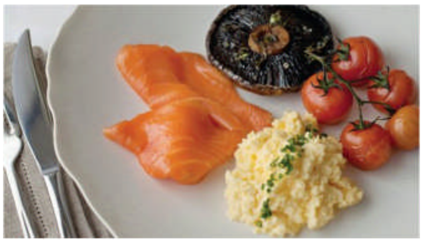

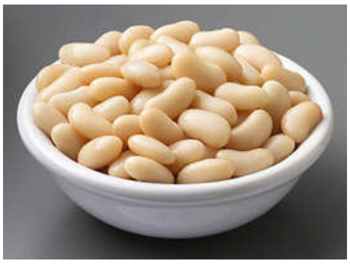

Attribution-NonCommercial-ShareAlike CC BY-NC-SA

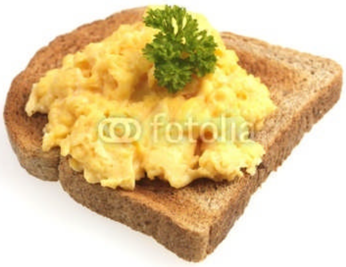

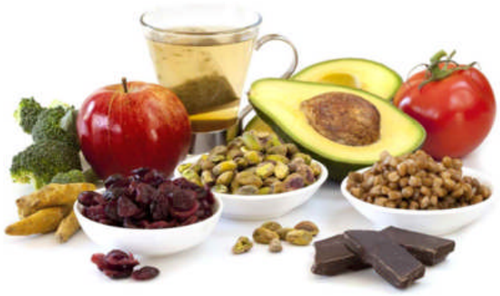

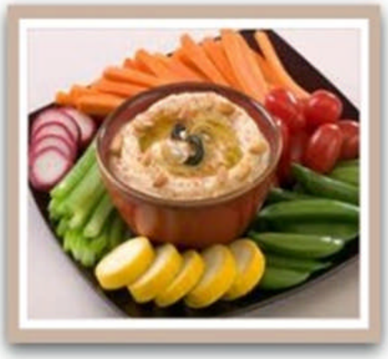

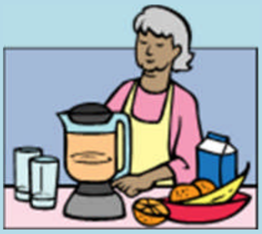

Breakfast smoothie recipe

Oatmeal

Almond, coconut, hemp seed or oat milk

Nuts and seeds

Some red fruit: strawberries, blueberries, raspberries You could add small amounts of other fruit: kiwis, apple, half a banana

Attribution-NonCommercial-ShareAlike CC BY-NC-SA

When and who we eat with

Like the well known saying: Breakfast like a king, lunch like a prince, dinner like a pauper!

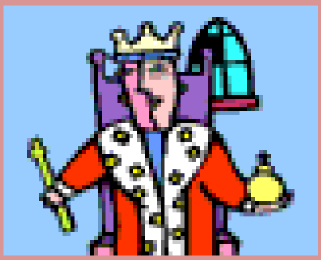

- It is important to start with a good breakfast
- Eat at regular times
- Try to avoid eating too late.

- We also discussed the value of eating together.

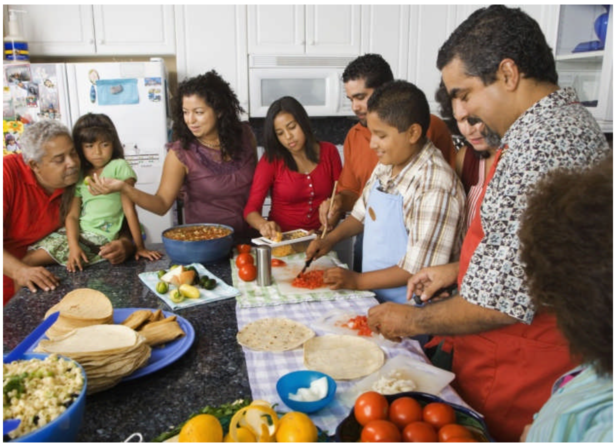

Attribution-NonCommercial-ShareAlike CC BY-NC-SA
